# Integrative Exploration of *Paenibacillus* sp. JSM-10 as a Potential Multi-Stress-Tolerant Microbial Inoculant for Sustainable Agriculture

**DOI:** 10.3390/ijms27094062

**Published:** 2026-04-30

**Authors:** Zhasmin Zhaksybek, Adel Sattarova, Ainur Akimbekova, Aldan Shamukhan, Irina Rukavitsina, Sailau Abeldenov, Anuar Rysbekovich Zhumakayev

**Affiliations:** 1 Laboratory of Molecular Biotechnology, National Center for Biotechnology, Astana 010000, Kazakhstan; 2 Department of Biotechnology and Microbiology, L.N. Gumilyov Eurasian National University, Astana 010000, Kazakhstan; 3 Microbiology Laboratory, A.I. Barayev Research and Production Centre for Grain Farming, Nauchny Set., Akmola Region 021601, Kazakhstan

**Keywords:** *Paenibacillus* sp., PGPB, antagonistic activity, glyphosate, stress tolerance, plant-microbe stress alleviation, molecular biotechnology

## Abstract

Abiotic stress factors, including drought and salinity, severely limit crop productivity worldwide. Furthermore, the extensive use of herbicides, such as glyphosate, disrupts beneficial soil microbiota, further impairing crop growth. Plant growth-promoting bacteria (PGPB) represent a sustainable and efficient strategy to enhance crop yields, particularly under unfavorable environmental and soil conditions. In this study, we characterized *Paenibacillus* sp. JSM-10, newly isolated from glyphosate-exposed agricultural soil, for its stress tolerance and plant growth-promoting potential, including its morphology examined using complementary microscopy techniques. The strain tolerated up to 0.5 g/L glyphosate, 15 g/L NaCl, and 100 g/L polyethylene glycol (PEG-6000) without significant growth inhibition (*p* > 0.05), demonstrating robust resilience to such multiple abiotic stresses. Beyond its tolerance, the strain exhibited several beneficial characteristics, including indole-3-acetic acid (IAA) synthesis, siderophore production, and inorganic phosphate solubilization. Furthermore, both living cells and culture filtrates of JSM-10 exhibited a positive trend toward enhancing buckwheat growth under normal and saline conditions, with effect sizes ranging from Hedges’ *g* = 0.56−0.92. In addition, JSM-10 exhibited antagonistic activity against a range of pathogenic microorganisms, including *Nigrospora oryzae*, *Bipolaris sorokiniana*, *Alternaria* spp., and *Escherichia coli*. Altogether, these characteristics highlight the *Paenibacillus* sp. JSM-10 strain and its culture filtrates as promising candidates for application in organic farming aimed at promoting plant growth and improving stress tolerance via plant–microbe interactions.

## 1. Introduction

Growing environmental pressures, notably pesticide pollution, water scarcity, and soil salinization, represent major limiting stress factors in modern agriculture [1]. Soil pollution by pesticide residues has emerged as a serious threat and has been recently reported as even the second major factor altering soil biodiversity [2]. This is especially concerning because pesticide pollution negatively impacts the abundance of beneficial plant-associated bacteria with plant growth-promoting characteristics [3]. A clear example is the widely utilized herbicide glyphosate N-(phosphonomethyl) glycine, which is repeatedly reported to inhibit a broad range of beneficial soil and plant microorganisms [4,5]. Such effects not only compromise microbial-mediated nutrient cycling but can also reduce crop productivity in glyphosate-treated soils. Salinity and drought also cause major abiotic stress conditions, significantly limiting crop yields. Saline conditions strongly affect plant growth by disturbing ionic and osmotic balance, altering redox status and cellular energy, as well as reducing photosynthesis [6]. Drought is another major constraint in agriculture, negatively affecting water balance, increasing the production of reactive oxygen species (ROS) [7], and causing average cereal production losses of up to 9–10% [8].

Altogether, pesticide exposure, salinity, and drought substantially constrain crop productivity, highlighting the need for advanced sustainable agricultural strategies capable of mitigating adverse abiotic conditions [9,10]. The application of environment-friendly methods, such as plant growth-promoting bacteria (PGPB), represents an effective strategy to maintain stable food production and mitigate the effects of abiotic stress factors [11,12]. PGPB enhance plant growth through multiple mechanisms, including inorganic nutrient solubilization, atmospheric nitrogen fixation, phytohormone production, and synthesis of bioactive metabolites that stimulate plant development [13]. Furthermore, stress-resilient PGPB strains represent robust biotechnological agents for sustainable agriculture, as they could enhance resistance to plant pathogens and improve crop yields under exposure to abiotic stress conditions [14].

Currently, PGPB include taxonomically diverse bacterial groups with distinct growth-promoting and protective mechanisms [15], including different strains of *Pseudomonas*, *Bacillus*, *Azotobacter*, and *Paenibacillus*. The ongoing description of new species continues to expand the diversity of promising PGPB. For example, *Paenibacillus peoriae*, first distinguished as a distinct species in 1993 (at that time as *Bacillus peoriae*) [16], has recently attracted increasing attention as a promising candidate for plant growth promotion, biocontrol, and biotechnological applications [17,18,19]. This species clearly demonstrates plant growth-promoting characteristics typical of *Paenibacillus*, including the production of growth-promoting substances, siderophore synthesis, and phosphorus solubilization [19], and exhibits remarkable antagonistic activity against pathogenic microorganisms, such as *Fusarium oxysporum*, *Bipolaris sorokiniana*, and *Alternaria alternata* [20]. These results highlight the potential of *Paenibacillus*-like strains to improve plant growth and productivity, suggesting their valuable potential in organic farming systems.

The *Paenibacillus* genus is increasingly recognized as a promising PGPB, yet its strain-specific metabolic potential, antagonistic activity, and growth stimulation across diverse crops remain insufficiently explored. In addition, tolerance of PGPB to environmentally relevant stress factors is often not comprehensively evaluated, limiting a reliable assessment of its field applicability. Therefore, the present study aimed to provide an extensive evaluation of a newly isolated glyphosate-tolerant *Paenibacillus* sp. JSM-10 strain and its multi-stress tolerance, plant-stimulating, and crop-protecting characteristics to assess its suitability as a robust microbial candidate for improving plant performance under adverse environmental conditions.

## 2. Results

### 2.1. Isolation, Identification, and Microscopic Evaluation of Paenibacillus *sp.* JSM-10

#### 2.1.1. Isolation of JSM-10 Strain

A bacterial isolate was successfully obtained from soil collected in a soft spring wheat field (cultivar “Aqmola 2”) in Kazakhstan. Following serial dilution and plating on selective medium amended with 1 g/L glyphosate, several morphologically distinct colonies were observed. Among them, one dominant isolate with consistent growth characteristics was considered a potential glyphosate-tolerant strain and selected for further analysis. The isolate formed circular, creamy-white colonies with smooth margins, indicating a typical phenotype of *Paenibacillus*-like bacteria.

#### 2.1.2. Identification of Glyphosate-Tolerant Strain JSM-10

**Figure 1:**
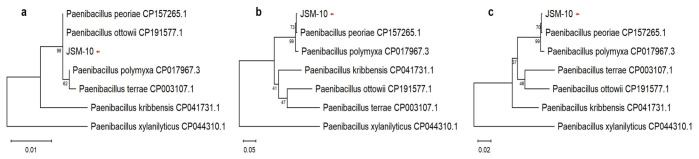
Phylogenetic analysis of strain JSM-10 and closely related *Paenibacillus* species based on different genetic markers. (**a**) Maximum Likelihood phylogenetic tree based on 16S rRNA gene sequences. (**b**) Maximum Likelihood phylogenetic tree based on *gyrA* gene (gyrA2 fragment) sequences. (**c**) Maximum Likelihood phylogenetic tree based on concatenated 16S rRNA and *gyrA* gene (gyrA2 fragment) sequences. Bootstrap values (1000 replicates) are shown at branch nodes. The strain JSM-10 is indicated with a red arrow.

Based on the sequence analysis of the 16S rRNA gene, the isolate was initially identified as belonging to the genus *Paenibacillus*; however, a precise species-level diagnosis could not be achieved through this marker alone. Consequently, sequences of the DNA gyrase subunit A (*gyrA*) gene and concatenated datasets of 16S rRNA and *gyrA* were also analyzed, indicating a close phylogenetic affiliation of the strain with *Paenibacillus peoriae* (Figure 1).

In addition to taxonomic markers (16S rRNA and two fragments of *gyrA*), subsequent amplification and sequencing of the *rho* gene further indicated the phylogenetic placement of the strain within the genus *Paenibacillus* and its close relationship to *P. peoriae*. This taxonomic placement is supported by high similarity scores in BLAST alignments, and the sequences were uploaded to the NCBI database (16SrRNA: PZ059921, *gyrA*: gyrA2: PZ241072, gyrA1: PZ287672, *rho*: PZ316596). Phylogenetic analysis of an amplified region of *gyrA* (gyrA1) and *rho* is given in the Supplementary Figures S1 and S2.

While the multi-marker approach indicates a close affinity to *P. peoriae*, the strain was conservatively designated as *Paenibacillus* sp. JSM-10 to reflect current genome-based (ANI/dDDH) requirements for species-level confirmation in this taxonomically complex genus.

#### 2.1.3. Microscopic Evaluation of *Paenibacillus* sp. JSM-10

**Figure 2:**
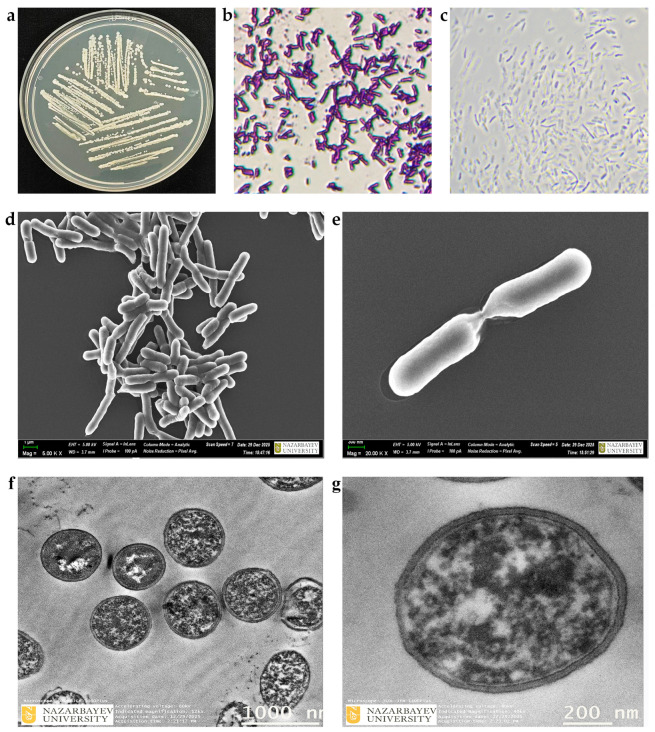
Morphological characterization of *Paenibacillus* sp. JSM-10. (**a**) Colony morphology of JSM-10 grown on YEG medium. (**b**) Gram-stained cells observed under light microscopy. (**c**) Phase-contrast microscopy image. (**d**) Scanning electron microscopy (SEM) image of clusters of rod-shaped cells; (**e**) SEM of high-magnification image of a dividing cell. (**f**) Transmission electron microscopy (TEM) image of intracellular ultrastructure. (**g**) TEM of intracellular components and cellular morphology.

Light and phase-contrast microscopy, along with scanning and transmission electron microscopy (SEM and TEM), were employed to characterize the Gram staining, bacterial morphology, cellular morphology, and cell structure of the isolate, respectively (Figure 2).

The cellular morphology of the bacterial isolate JSM-10 grown on YEG medium (Figure 2a) represented typical *Paenibacillus* morphology and was further characterized using multiple microscopy techniques. Light microscopy (Figure 2b) and phase-contrast microscopy (Figure 2c) revealed Gram-positive staining and showed uniform *Paenibacillus*-type rod-shaped cells, respectively. Advanced microscopy, such as SEM, provided detailed images of the cell surface and overall shape, revealing smooth, rod-shaped cells with intact cell walls (Figure 2d,e). TEM analysis allowed observation of internal structures, such as cytoplasm density, and also highlight the typical ultrastructure of the isolate (Figure 2f,g). Together, these analyses demonstrated a comprehensive view of the isolate’s morphology and growth patterns of the strain at both the cellular and subcellular levels.

### 2.2. Abiotic Stress Tolerance of Paenibacillus *sp.* JSM-10

#### 2.2.1. Evaluation of Glyphosate and NaCl Tolerance

**Figure 3:**
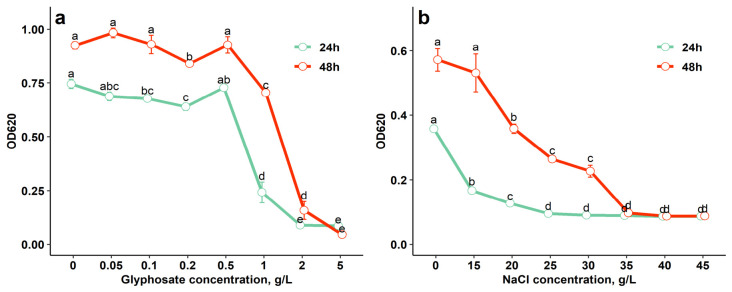
Growth response (OD_620_) of *Paenibacillus* sp. JSM-10 under (**a**) glyphosate and (**b**) NaCl stress factors, across concentration gradients. Different letters indicate statistically significant differences between treatments (*p* < 0.05, one-way ANOVA followed by Tukey’s HSD test).

The growth of JSM-10 was not significantly reduced in the glyphosate concentration range of 0.05–0.5 g/L (Figure 3a) compared to the control (0 g/L) (*p* > 0.05), demonstrating its considerable tolerance to glyphosate.

The OD_620_ values of JSM-10 were significantly reduced only at higher glyphosate concentrations (1–5 g/L; *p* < 0.05). The strongest inhibition was observed at 2–5 g/L after both 24 h and 48 h (*p* < 0.05), indicating a concentration-dependent inhibitory effect of glyphosate (Figure 3a).

The growth of bacterial strain JSM-10 remained stable at NaCl concentrations of up to 15 g/L (Figure 3b). Although growth at this concentration was lower than the control without NaCl after 24 h (*p* < 0.05), no significant differences were observed at 48 h (*p* > 0.05), indicating growth recovery under saline stress. Cell density gradually decreased at 20–30 g/L (*p* < 0.05), while complete inhibition occurred at 40–45 g/L at both 24 and 48 h. PGPB capable of growth at NaCl concentrations of 1–5% (10–50 g/L) are classified as having low halotolerance [21]; accordingly, *Paenibacillus* sp. JSM-10 can be considered a salt-tolerant strain.

Interestingly, measurements at 24 and 48 h revealed distinct temporal patterns in the stress tolerance of strain JSM-10. Under glyphosate exposure, growth remained comparable between the two time points, whereas under NaCl stress, the strain exhibited improved growth after 48 h relative to 24 h, particularly at 15–30 g/L.

#### 2.2.2. Examination of Drought Tolerance, Optimal Temperature Range, and Resistance to Heat-Shock Exposure

**Figure 4:**
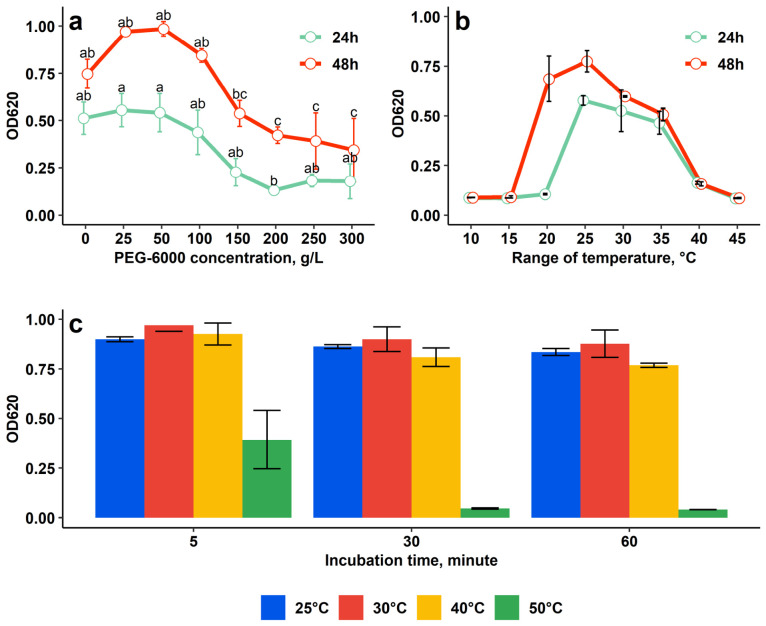
Drought tolerance assay (**a**), optimal temperature range (**b**), and resistance to heat-shock treatment (**c**) of glyphosate-tolerant *Paenibacillus* sp. JSM-10. Different letters indicate statistically significant differences between treatments (*p* < 0.05, one-way ANOVA followed by Tukey’s HSD test).

Growth of JSM-10 under PEG-6000-induced drought stress remained stable at 25–100 g/L (*p* > 0.05) and was significantly inhibited only at ≥150 g/L (*p* < 0.05) (Figure 4a). These findings indicate strong osmotic stress tolerance, as growth occurred at concentrations exceeding the 40 g/L PEG level previously associated with drought tolerance [22].

Evaluation of the optimal temperature range revealed that cell density remained low at 10–15 °C, showed the highest growth at 20–35 °C, and declined at 40 °C, with minimal growth observed at 40 and 45 °C (Figure 4b). The growth pattern was the same in both 24 and 48 h incubation, except 20 °C, at which low cell density was observed in the 24 h measurement, with a remarkable increase after a longer, 48 h incubation. Based on these findings, the optimal temperature levels for JSM-10 fall in the range of 20–35 °C.

Heat-shock testing performed to evaluate the tolerance of JSM-10 to sharp temperature changes during vegetation time in vivo showed high survival across tested temperatures, except 50 °C (Figure 4c). Bacterial recovery at 25 °C, 30 °C, and 40 °C was comparable to the control across all exposure times.

### 2.3. PGPB Characteristics of JSM-10

#### 2.3.1. Indole-3-Acetic Acid (IAA) Synthesis, Phosphorus Solubilization, and Siderophore Production of *Paenibacillus* sp. JSM-10

**Figure 5:**
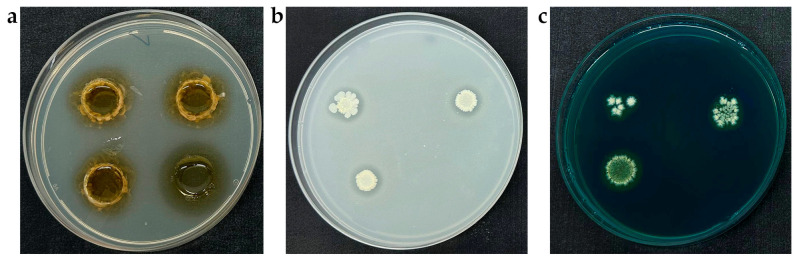
Plant growth-promoting traits of *Paenibacillus* sp. JSM-10: (**a**) IAA production on YEG medium, (**b**) phosphate solubilization on Pikovskaya agar, (**c**) siderophore production on CAS blue agar medium.

*Paenibacillus* sp. JSM-10 demonstrated multiple plant growth-promoting traits, including IAA synthesis, phosphate solubilization, and siderophore production (Figure 5). IAA production was confirmed by the development of a distinct pink coloration upon addition of Salkowski’s reagent to cultures grown in YEG supplemented with 0.1 g/L L-tryptophan (Figure 5a). Quantitative analysis revealed an IAA concentration of 70.3 µg/mL in the culture supernatant after three days of incubation.

Phosphate solubilization was observed on Pikovskaya agar (Figure 5b), where clear halo zones formed around the colonies, indicating the microbial dissolution of insoluble calcium phosphate (CaHPO_4_). On the 10th day of incubation, the solubilization index ranged from 1.30 to 1.38 and was highest at an inoculum density of 1 × 10^6^ CFU/mL. An additional PGPB characteristic, siderophore formation, was detected on Chrome Azurol S (CAS) agar by the appearance of a defined orange halo surrounding the colonies, resulting from iron chelation from the CAS-Fe^3+^ complex (Figure 5c).

#### 2.3.2. The Effect of *Paenibacillus* sp. JSM-10 and Its Cell-Free Culture Filtrates (CCFs) on Buckwheat Growth

**Figure 6:**
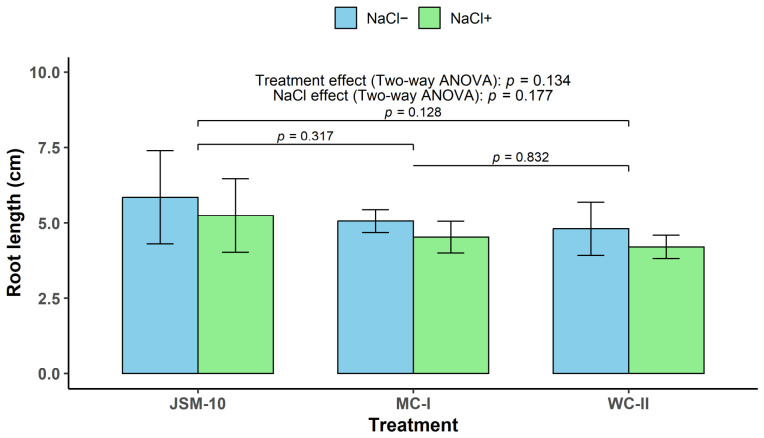
Root length of buckwheat under normal (NaCl^−^) and saline (NaCl^+^) conditions. **JSM-10** is the combined effect of living culture of *Paenibacillus* sp. JSM-10 strain and its CCF; **MC-I** represents CCF from non-inoculated medium; **WC-II** is distilled H_2_O. Brackets indicate *p*-values for pairwise comparisons between treatments across NaCl conditions based on two-way ANOVA followed by Tukey’s HSD test.

JSM-10 exhibited a positive trend toward enhancing buckwheat root length compared with both applied medium and water controls (Figure 6). Under non-saline conditions (NaCl^−^), roots inoculated with JSM-10 reached 5.85 cm, resulting in an increase of up to 15.51 and 21.68% compared to the medium control (MC-I) and water control (WC-II), respectively.

A similar growth-promoting tendency of *Paenibacillus* sp. JSM-10 on the buckwheat root length was observed with the exposure to saline conditions (Figure 6). Under NaCl+, JSM-10 maintained its promoting trend on the root length, resulting in a higher root length (5.25 cm) relative to both controls: MC-I (up to 15.82%) and WC-II (up to 24.72%).

Two-way ANOVA revealed no statistically significant effects of NaCl exposure (*p* = 0.177) or treatment type (*p* = 0.134) on buckwheat root length. Post hoc Tukey’s HSD comparisons also showed no significant differences among treatments. However, the *p*-values indicated a decreasing tendency from the control treatments toward the bacterial inoculation. While the comparison between the two control variants (MC-I compared to WC-II) yielded a *p*-value of 0.83, the comparisons between JSM-10 and the MC-I and WC-II resulted in *p*-values of 0.32 and 0.13, respectively.

Interestingly, the growth-promoting tendency of JSM-10 on buckwheat roots was consistent across treatments, showing increases of 15.51–15.82% and 21.68–24.72% relative to the two controls (MC-I and WC-II), regardless of non-saline (NaCl^−^) and saline (NaCl^+^) conditions, respectively. The effect sizes (Hedges’ *g*) for JSM-10 compared to treated samples with MC-I and WC-II under normal and saline conditions were 0.56 [95% CI: −0.80, 1.86] and 0.66 [95% CI: −0.73, 1.98], as well as 0.61 [95% CI: −0.76, 1.92] and 0.92 [−0.54, 2.30], respectively. Such a non-significant yet consistent positive growth trend under both normal and saline conditions suggests that JSM-10 may possess plant growth-promoting potential and may contribute to stress mitigation.

### 2.4. Antagonistic Potential of Glyphosate-Tolerant JSM-10 Against Different Pathogenic Species

**Figure 7:**
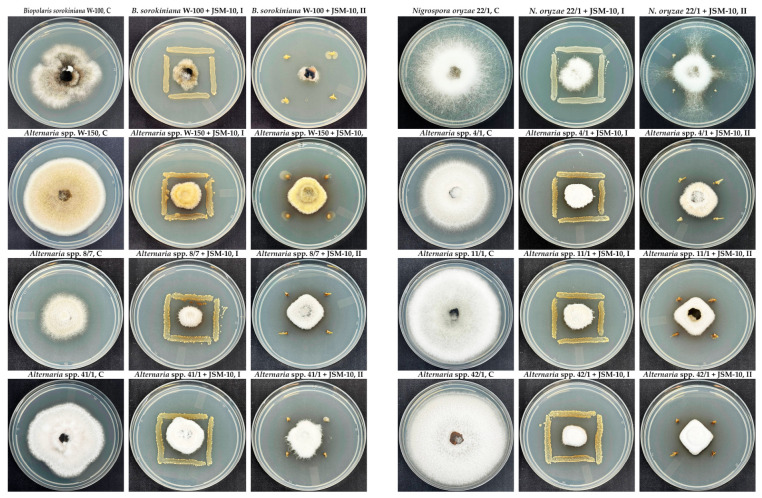
Antagonistic activity of *Paenibacillus* sp. JSM-10 strain against phytopathogenic fungi *B. sorokiniana*, *N. oryzae*, and 6 strains of *Alternaria* spp. C is the control (growth in the absence of JSM-10), bacterial-treated plates are indicated as “+JSM-10”; I and II represent high- and low-inoculum treatments, respectively.

The antagonistic activity of bacterial strain JSM-10 was evaluated against the phytopathogenic fungi *B. sorokiniana*, *N. oryzae*, and *Alternaria* spp. on YEG medium. The strain demonstrated clear inhibitory effects on fungal pathogens, as evidenced by remarkably reduced mycelial growth compared to the control (Figure 7).

Both tested low- and high-inoculum methods inhibited fungal growth, confirming a strong antagonistic activity of JSM-10 towards all tested fungal strains. A visible color change of the medium between JSM-10 and *Alternaria* strains indicated that possible production of diffusible metabolites occurred during co-culture. The observed inhibition zones indicated strong antagonistic potential, suggesting the production of antifungal metabolites or competition for nutrients.

Quantitatively, the inhibition rate of JSM-10 in dual-culture assays varied between 64.2% towards *B. sorokiniana*, 71.2% to *N. oryzae*, and 72.1% against *Alternaria* spp. after 7 days of incubation (Table 1).

The observed inhibition, regardless of either low- or high-inoculum treatments, indicated a dose-independent mode of antagonistic activity of JSM-10. Except for *Alternaria* 8/7 with low-inoculum samples (36.8%), the growth of all tested fungal phytopathogens was inhibited by more than 50% on all bacterial-treated plates. The results confirm the remarkable potential of JSM-10 for application as a promising antagonistic inoculant to protect plant growth.

Additionally, the bacterial strain JSM-10 exhibited pronounced antagonistic activity against *E. coli* DH5α. Inhibition was observed on YEG agar plates inoculated with *E. coli*, where JSM-10 produced distinct, clear inhibition zones, indicating antimicrobial activity (Supplemental Figure S3). Kinetic assays performed to verify the presence of active metabolites revealed that, although 50% CCF obtained from non-inoculated medium (MM + Ala or MM + Glu) reduced cell density by only 6.80–12.56%, CCF (50%) obtained from JSM-10 grown in MM supplemented with L-alanine or glucose inhibited the growth of *E. coli* by up to 45.13–45.40%, respectively (Table 1). These findings suggest that extracellular metabolites produced by JSM-10 contribute substantially to its antibacterial activity.

### 2.5. Molecular Detection of Genes Associated with Plant Growth Promotion and Stress Tolerance

**Figure 8:**
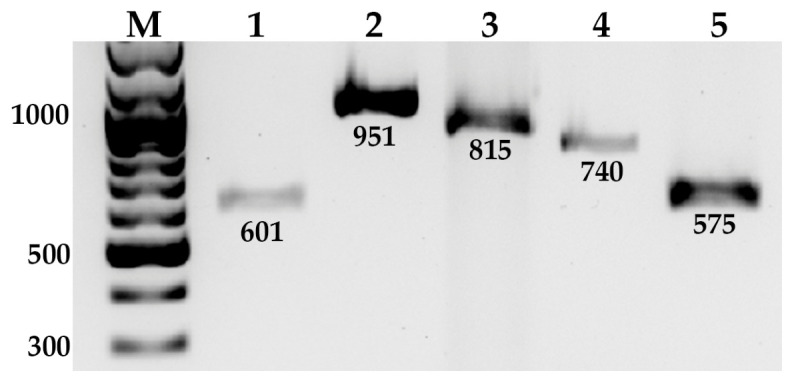
PCR-based detection of taxonomic and functional genes in *Paenibacillus* sp. JSM-10. Lane M: DNA marker (SM0333, ThermoFisher Scientific); lane 1–5: *gcd*, *ipdC*, *thiO*, *ectA*, and *groL*, respectively.

PCR amplification was performed to assess the presence of genes associated with plant growth-promoting traits and stress tolerance in strain JSM-10 (Figure 8).

PCR products of the expected sizes were obtained for *gcd*, *ipdC*, *thiO*, *ectA*, and *groL*, indicating the presence of genes putatively associated with IAA biosynthesis, phosphate solubilization, and stress-related functions. Bands corresponding to the expected amplicon sizes were observed for all targets, indicating the presence of genes associated with plant growth-promoting traits and stress tolerance in strain JSM-10.

## 3. Discussion

### 3.1. Plant Growth-Promoting (PGP) Potential and Mechanisms of Paenibacillus

#### 3.1.1. *Paenibacillus* as a Promising PGPB Genus

A promising strategy to enhance soil quality, increase crop yields, and minimize the effects of ecological factors is the integration of organic farming relying on environmentally friendly methods, such as beneficial soil microflora or PGPB [13]. PGPB are considered cost-effective and easily accessible biological tools for mitigating both biotic and abiotic stresses. Therefore, integrating PGPB into crop production systems supports long-term sustainability and helps to preserve soil biodiversity by reducing dependence on chemical fertilizers [14]. *Paenibacillus* spp. represent an effective and promising PGPB genus, numerous species of which were reported to exhibit versatile plant growth–promoting and antagonistic properties (Table 2).

In addition to their PGP traits, the strains of *Paenibacillus* are known for their efficient ecological compatibility. For many introduced *Paenibacillus* strains used as PGPR/biocontrol inoculants, field and soil studies show no large, sustained disruption of resident bacterial communities [35]. As an autochthonous isolate from the Kazakh steppe, the indigenous origin of JSM-10 represents a logically compatible candidate for the local environment. These factors position the strain as a promising candidate for further biosafety and ecological impact assessments prior to large-scale field applications.

#### 3.1.2. PGP Mechanisms of *Paenibacillus* sp. JSM-10 Strain

Overall, these studies demonstrate diverse PGPB traits on different crops across members of the genus *Paenibacillus*. One of the most important PGPB traits exhibited by *Paenibacillus* spp. is the production of the phytohormone IAA, a major auxin that directly influences root architecture, cell division, and nutrient acquisition [36]. In *Paenibacillus* species, IAA production is commonly associated with L-tryptophan-dependent pathways [37,38], which is consistent with the experimental conditions used in this study.

Siderophore production is another key trait of *Paenibacillus* spp., enhancing rhizosphere iron mobilization and nutrient uptake under iron limitation [39]. For example, *Paenibacillus illinoisensis* YZ29 increased rhizosphere iron availability by 1.8-fold and shoot biomass by 23%, demonstrating a direct link between bacterial siderophores and improved iron nutrition and growth [29]. Phosphorus solubilization is also a key trait of *Paenibacillus* spp., allowing plants to access otherwise unavailable phosphate pools in the rhizosphere. Inoculation of wheat with the phosphate-solubilizing *Paenibacillus* sp. B1 significantly increased soil available phosphorus by about 9% and shoot biomass by approximately 30% compared with non-inoculated controls, demonstrating that bacterial P solubilization can partially substitute for chemical phosphorus fertilizers [24]. Genomic analyses of *Paenibacillus sonchi* SBR5 revealed the presence of conserved glucose-1-dehydrogenase and gluconate dehydrogenase genes, supporting its gluconate-mediated phosphate-solubilization capacity [40].

The glyphosate-tolerant JSM-10 demonstrated key plant growth-promoting traits characteristic of the genus *Paenibacillus*, including IAA synthesis, siderophore production, and phosphorus solubilization (Figure 5). The co-occurrence of these traits has been widely reported in *Paenibacillus* strains and is considered an important factor contributing to their multifunctional role in plant growth promotion and biocontrol [26,41]. Reported specific structural genes and biosynthetic clusters—such as the *ipdC* gene encoding indole-3-pyruvate decarboxylase for auxin (IAA) production, and non-ribosomal peptide synthetase (NRPS) operons governing siderophore assembly—provide a concrete genetic framework. The presence of these dedicated loci elucidates the underlying molecular background driving the multiple plant growth-promoting (PGP) benefits, including enhanced root development and iron acquisition, observed in *Paenibacillus* sp. strain JSM-10 [42]. Altogether, these results demonstrate key plant growth-promoting traits of *Paenibacillus* sp. JSM-10, including phytohormone production, phosphate solubilization, and siderophore production, supporting its potential as a microbial inoculant.

#### 3.1.3. Enhancement of Buckwheat Growth by JSM-10 Under Normal and Saline Conditions

Buckwheat is an important pseudocereal gluten-free crop widely consumed worldwide with high nutritional value and numerous health benefits [43,44]. It contains bioactive components, such as peptides, flavonoids, phenolic acids, fagopyritols, and fagopyrins, which makes it attractive both for human nutrition and for diversified cropping systems [45]. Despite its increasing global importance, research on PGPB for this crop remains limited, and reports on *Paenibacillus*–buckwheat interactions are particularly scarce, highlighting the relevance of evaluating new strains for this valuable crop.

The living culture of *Paenibacillus* sp. JSM-10 strain combined with the obtained from liquid culture CCF stimulated buckwheat root length up to 21.68-24.72% under normal and saline conditions, respectively (Figure 6). These findings indicate that the total bacterial inoculant, including both the cells and their secreted bioactive compounds, may contribute to increased plant growth. Although the differences were not statistically significant, the results suggest a tendency toward increased root length in plants treated with JSM-10. The absence of statistically significant differences is likely related to the relatively small sample size (*n* = 3 per treatment).

Root length is one of the most commonly used and informative indicators for evaluating PGPB effects and is suggested as a primary criterion for selecting promising strains [46]. The observed root-length increase by JSM-10 supports its further evaluation as a promising PGPB candidate. This integrated effect is consistent with reports on this genus, which is known for producing a wide range of metabolites that improve nutrient availability and stimulate root development [47].

While members of *Paenibacillus* genus demonstrated different plant-growth-stimulating mechanisms on a wide range of crops (Table 2), information on the effect of *Paenibacillus* species on buckwheat growth is limited. Seed bacterization with phosphate-solubilizing *P. polymyxa* KB balanced a phosphorus shortage in buckwheat [48]. The observed growth-promoting trend of strain JSM-10 broadens the portfolio of crops previously reported to be positively influenced by *Paenibacillus* sp.

### 3.2. Antagonistic Activity and Biocontrol Potential

In addition to auxin production and nutrient mobilization, *Paenibacillus* strains can suppress plant pathogens through diverse antimicrobial metabolites and induce plant defenses [19]. Members of this genus produce secondary metabolites, including nonribosomal peptides, lipopeptides, and volatile organic compounds (VOCs), which contribute to biocontrol activity [18]. Genomic analyses of biocontrol-active *P. peoriae* strains revealed gene clusters encoding nonribosomal peptide synthetases (NRPSs) and polyketide synthases (PKSs) that underpin production of these antagonistic metabolites, which can also trigger plant defense signaling [17]. Our JSM-10 strain inhibited several pathogens (Table 1), including *B. sorokiniana* (64.2%), *N. oryzae* (72%), and *Alternaria* spp. (72%) and *E. coli* (45.4%), consistent with the broad-spectrum antimicrobial activity associated with these molecular mechanisms. These findings suggest that both the living cells of JSM-10 and their bioactive metabolites contribute to pathogen suppression and may enhance systemic resistance in crops, supporting its potential as a multifunctional biocontrol agent.

Overall, JSM-10 exhibited strong antagonistic activity against *E. coli* and multiple fungal pathogens (*B. sorokiniana*, *N. oryzae*, and *Alternaria* spp.), indicating broad-spectrum biocontrol potential.

### 3.3. Eco-Physiological Characterization of JSM-10

#### 3.3.1. Tolerance to Glyphosate Exposure

The global trends suggest that for successful field applications, PGPB require sufficient stress tolerance because beneficial effects can be limited by abiotic stress factors, such as soil contamination, salinity, and drought conditions. Therefore, PGPB need to be tested for systematic, multi-factor stress profiling in vitro prior to agriculturally relevant conditions [49]. For example, glyphosate is one of the most widely used herbicides worldwide and is known to exert toxic effects on non-target soil microorganisms [50], disrupting the growth, metabolism, and plant growth-promoting activities of many beneficial plant-associated fungi and bacteria [51]. Studies have shown that exposure to glyphosate can reduce microbial diversity, inhibit beneficial bacteria such as *Pseudomonas* and *Bradyrhizobium japonicum* [5], and impair their production of plant growth-promoting compounds like indole-3-acetic acid (IAA) and siderophores. The extensive utilization of this herbicide led to its dispersion and accumulation in both terrestrial and aquatic environments, heightening its potential hazards to the endemic soil microbiome and introduced PGPB [52,53].

In our studies, JSM-10 tolerated glyphosate up to 0.5 g/L without significant growth inhibition (*p* > 0.05), compared to the control without glyphosate (Figure 3). These findings are particularly relevant for agricultural applications since soils are frequently contaminated with glyphosate residues worldwide. Such residues can impact PGPB negatively, and, therefore, glyphosate tolerance of potential PGPB should be evaluated prior to in vivo applications.

Glyphosate showed variable levels of soil contamination but overall represented the largest contribution to total pesticide residues in soils, with a maximum concentration of 2.05 mg/kg [54]. Therefore, the glyphosate tolerance of JSM-10 examined in this study demonstrated clear tolerance and growth maintenance under herbicide stress up to 0.5 g/L, remarkably above the concentrations found in the field. This highlights its potential application as a glyphosate-tolerant PGPB to support sustainable crop production in agricultural systems, including glyphosate-polluted environments. Moreover, glyphosate exposure has been associated with endocrine disruption and genotoxic effects [55,56]. The utilization of glyphosate-tolerant PGPB is therefore a foundational step for agricultural applications in contaminated soils. Such strains can maintain their growth-promoting activities under herbicide stress and could represent potential candidates for future studies on glyphosate bioremediation to reduce environmental and health risks.

Mechanistically, the resilience of *Paenibacillus* to xenobiotic stress is often linked to a highly versatile genomic repertoire encoding stress-responsive enzymes, such as oxygenases and dehydrogenases, which play key roles in the degradation of complex environmental pollutants [42]. While the specific gene expression networks governing glyphosate tolerance in JSM-10 require further genetic characterization, these inherent genetic features highlight the genus’s capacity to tolerate chemical toxicity.

#### 3.3.2. Tolerance to Salinity and Drought Stress

The *Paenibacillus* sp. JSM-10 was isolated from a semi-arid steppe soil environment defined by severe drought and fluctuating salinity. Such regions remain relatively under-explored for the discovery of stress-resilient PGPB capable of maintaining crop performance under adverse environmental conditions.

The JSM-10 strain demonstrated notable resilience to subsequent adverse abiotic stress factors, maintaining growth at moderate salt concentrations up to 15 g/L (Figure 3). These observations are in line with previous findings on *P. polymyxa*, which tolerated NaCl concentrations of 0–2%, with only gradual growth inhibition at 3–4%, demonstrating good salt tolerance in moderately saline soils [57]. In other studies, *P. polymyxa* strains also grew well at NaCl concentrations up to 4%, highlighting their ability to survive and remain active under saline conditions, further supporting their potential to enhance plant growth in salt-affected soils [58].

Furthermore, JSM-10 demonstrated robust osmotic stress resistance up to 150 g/L PEG 6000 and in optimal temperatures of 20–35 °C, and full recovery after prolonged heat-shock exposure up to 40 °C (Figure 4). Our findings are in accordance with previously published reports where members of the genus *Paenibacillus* have been increasingly recognized for their role in enhancing plant tolerance to drought stress. For example, *P. polymyxa* CR1 was shown to prime plants for drought by inducing dehydration-responsive genes such as RD29A and RD29B, thereby improving plant performance under water-limited conditions [59].

Importantly, drought mitigation by PGPB is often manifested not through enhanced accumulation of stress metabolites but rather through a reduction in stress-induced physiological responses. Accordingly, inoculation with Firmicutes, including *Paenibacillus stellifer*, was reported to significantly reduce the accumulation of osmolytes (proline and glycine betaine) and the activities of antioxidant enzymes (CAT and SOD) under moderate and severe drought stress, indicating effective stress mitigation. Similar trends observed in the present study suggest that this strain contributes to drought tolerance by stabilizing plant physiological status and reducing oxidative and osmotic stress, supporting the potential of *Paenibacillus* species as drought-mitigating PGPB [60].

At the molecular level, the environmental resilience of *Paenibacillus* is largely supported by the induction of stress-responsive genes regulating exopolysaccharide (EPS) biosynthesis—which acts as a physical biofilm barrier against osmotic shock and desiccation—and the synthesis of protective osmolytes [42]. Beyond the induction of exo-polysaccharide (EPS) biosynthesis—which forms a protective biofilm barrier against desiccation—*Paenibacillus* species leverages the synthesis of specific osmolytes, such as proline and trehalose, to stabilize cellular osmotic potential and protect plant enzymes under salt and water deficits [35].

Altogether, these findings, including tolerance up to 150 g/L PEG, growth under 20–35 °C, and recovery after exposure to heat-shock treatment, confirmed the drought tolerance potential of JSM-10, expanding its possible utilization under dry field conditions with sudden temperature fluctuations.

### 3.4. Ultrastructural Characterization of Cell Morphology and Structure

Microscopic characteristics of *Paenibacillus* members were reported previously, primarily based on individual techniques such as Gram staining or scanning electron microscopy [20]. However, a comprehensive morphological characterization integrating multiple microscopy approaches remains limited. In the present study, we provide a combined analysis of colony morphology, cellular structure, and ultrastructural features of *Paenibacillus* sp. JSM-10 using complementary microscopic techniques. This multi-scale evaluation provides an expanded understanding of its colony morphology, cellular organization, and ultrastructural features of the species, and also supports robust taxonomic and functional characterization of the isolate. The full collection of obtained microscopic images, including uncropped SEM and TEM presented in Figure 2, can be seen in Supplementary Materials (Supplementary Figures S4–S11).

### 3.5. Molecular Detection of Genes Putatively Involved in PGP and Stress Tolerance of JSM-10

To provide a molecular context for the observed phenotypic traits, selected genes associated with plant growth promotion and abiotic stress tolerance were examined in strain JSM-10. The targeted genes and their reported biological functions are summarized in Table 3.

The presence of *ipdC* and *gcd* correlates with the observed levels of IAA production and phosphate solubilization, respectively, while the identification of *thiO* supports the strain’s ability to tolerate and potentially degrade glyphosate. Furthermore, the detection of *ectA* and *groL* suggests an inherent genetic capacity for osmoprotection and proper protein folding, explaining the strain’s resilience under saline and thermal stress. While PCR-based detection of genes associated with plant growth-promoting traits and stress response supports the observed phenotypic characteristics of strain JSM-10, functional expression of these genes requires further investigation.

### 3.6. Potential Agricultural Applications, Current Limitations, and Future Perspectives

The successful agricultural application of promising PGPB requires a rigorous, multi-stage strategy. This roadmap consists of several subsequent steps, including the isolation and characterization of the beneficial inoculant, laboratory and greenhouse evaluations, ecological safety assessments, and finally, field trials and product certification [49].

The findings presented in this study establish the foundational stage of this strategy, providing the necessary evidence for the potential implementation of *Paenibacillus* sp. JSM-10. This strain was isolated from semi-arid steppe soil, which remains relatively underexplored as a source of stress-resilient plant growth-promoting bacteria. Tolerance to glyphosate exposure, salinity, and drought conditions highlights the potential of *Paenibacillus* sp. JSM-10 to enhance crop performance under environmental stress. Its resilience, together with the ability to induce systemic resistance and support plant growth, underscores its promise as a biological tool for sustainable and stress-resilient agriculture.

Such integrative characterization presented in the current study represents the essential foundational phase of a structured discovery-to-application pipeline. The following steps for agricultural applications include several aspects that remain to be addressed within the established evaluation pipeline for PGPB. Further genetic and molecular analyses are required to elucidate the mechanisms underlying stress tolerance and plant growth promotion. Longer-term greenhouse studies and detailed ecological safety assessments, including evaluation of potential effects on native rhizosphere microbiota, are necessary to ensure ecological compatibility. Subsequent field validation across diverse soil types and crop systems is required to confirm the strain’s efficiency under real agricultural conditions. These future studies will build upon the strong foundational dataset presented here and support the further development of strain JSM-10 for sustainable agricultural applications.

## 4. Materials and Methods

### 4.1. Isolation, Molecular Identification, and Microscopic Observation of Paenibacillus *sp.* JSM-10

#### 4.1.1. Isolation of Glyphosate-Tolerant JSM-10

The previously reported strategy [64] was applied with minor modifications. The soil sample was collected from a field of soft spring wheat (*Triticum aestivum* L.) previously treated with glyphosate-type herbicides (“Uragan Forte” and “Faraon Gold”). Soil suspension was prepared by mixing 5 g of soil in 40 mL physiological saline (0.9% NaCl), and 50 µL of a subsequent 1:10 dilution was spread on solid PMM medium (g/L: (NH_4_)_2_SO_4_ 4.49; K_2_HPO_4_ 1.5; MgSO_4_·7H_2_O 0.2; agarose 20) [65]. PMM was supplemented with 1 g/L glyphosate formulation (“Uragan Forte”, 500 g/L; Syngenta, Switzerland) as the sole carbon source. Nystatin and fluconazole (0.1 g/L each) were added to suppress fungal growth. Plates were incubated at 25 °C for 7 days. Colonies that appeared on PMM-glyphosate medium were considered glyphosate-tolerant bacteria, and individual colonies were purified by repeated streaking on YEG agar medium (g/L: yeast extract 5; glucose 10; agar 15), which was also used for routine maintenance of the purified isolate JSM-10.

#### 4.1.2. Molecular Identification and Detection of Functional Genes in JSM-10

Overnight bacterial culture was adjusted to 1 × 10^7^ CFU/mL in 50 μL double-distilled water (DDW) and used as a DNA template. Polymerase Chain Reaction (PCR) was performed using primer pairs and amplification programs, summarized in Table 4.

The PCR master mix for 16S rRNA (50 µL) consisted of the following components (final concentrations): 1 µL DNA template, 0.2 mM dNTPs, 1 µL Phusion Polymerase, 1 × HF buffer, 0.4 µM each primer; the final volume was adjusted with sterile MilliQ water. PCR was performed in a T100 Thermal Cycler (Bio-Rad, Singapore). The PCR products were visualized by 1% agarose gel electrophoresis and subsequently submitted for Sanger sequencing using an external service (National Scientific Shared Laboratory of Biotechnology, National Center for Biotechnology). All reagents were of molecular biology grade and purchased from New England Biolabs.

Two fragments (gyrA1 and gyrA2) of the *gyrA* gene were employed as a secondary molecular marker [67] to ensure accurate phylogenetic placement of strain JSM-10. The primers used in this study were designed based on multiple sequence alignment of *gyrA* gene sequences from representatives of the genus *Paenibacillus* using Vector NTI. Conserved regions were selected as primer binding sites, while the internal amplified fragment contained variable positions suitable for species-level discrimination after sequencing.

All amplification and post-PCR processing steps, including sequence assembly, were identical for both regions, except for the annealing temperature at 62 °C for the gyrA2 reaction (Table 4). The PCR master mix (25 µL) consisted of the following components (final concentrations): 1 µL DNA template, 0.2 mM dNTPs, 1 µL Taq polymerase, 1 × Taq buffer, 0.4 µM each primer; the final volume was adjusted with sterile MilliQ water. PCR was performed in a T100 Thermal Cycler.

Additionally, the *rho* gene (transcription termination factor) was amplified and sequenced to further support molecular identification of strain JSM-10 [18]. Primer design, PCR master mix composition, and post-PCR processing were identical to those used for the *gyrA* fragments.

Raw sequencing chromatograms obtained after Sanger sequencing were visually inspected and trimmed to remove low-quality regions. Forward and reverse reads were assembled into consensus sequences using Vector NTI Advance 11.0 software. The resulting contigs were checked to ensure correct base calling and absence of ambiguous nucleotides.

The obtained nucleotide sequences were compared with publicly available sequences in the NCBI GenBank database using the BLASTn algorithm in order to determine the closest homologs and confirm taxonomic affiliation. Reference sequences showing the highest similarity scores were selected for subsequent phylogenetic analysis.

Multiple sequence alignment of the obtained sequences together with the selected reference sequences was performed using the MUSCLE algorithm. For additional phylogenetic reconstruction, sequences of the 16S rRNA gene and *gyrA* gene fragments were concatenated, resulting in a combined alignment of 1390 and 1118 bp with gyrA1 and gyrA2, respectively. Concatenation of 16S rRNA and *rho* gene sequences resulted in an alignment of 1191 bp. Phylogenetic trees were constructed using the Maximum Likelihood (ML) method with the best-fit nucleotide substitution model determined automatically by the MEGA software (version 12.1.2). The robustness of the inferred phylogenetic relationships was evaluated by bootstrap analysis with 1000 replicates.

For amplification of genes associated with plant growth-promoting traits and stress tolerance (Table 4), specific primers were designed for *ipdC* (IAA synthesis), *gcd* (phosphate solubilization), *thiO* (glyphosate degradation), and *groL* and *ectA* (osmotic and saline stress tolerance). Primer design, PCR master mix composition, and amplification conditions were generally consistent with those used for *gyrA* and *rho*, except for *groL* and *ectA*, for which a modified reaction mixture was applied. The PCR master mix for *groL* and *ectA* contained 0.2 µM of each primer, 0.2 mM dNTPs, 1× Q5 buffer, 0.5 µL Q5 polymerase, 4% DMSO, and 1 µL DNA template in a final volume of 25 µL.

PCR amplicons were separated on 1% agarose gels (90 V, 40 min) and visualized using a GelDoc Go Imaging System (Bio-Rad, USA). The presence of target genes was assessed based on the expected amplicon size.

#### 4.1.3. Microscopic Evaluations of Glyphosate-Tolerant *Paenibacillus* sp. JSM-10

Bacterial morphology was examined after cultivation of JSM-10 on YEG for 2 days. Typical morphological characteristics, such as color, form, and size, were recorded based on visual observations.

The Gram reaction was performed using a fresh bacterial culture in accordance with the manufacturer’s instructions for the Gram Stain Kit (HiMedia, Maharashtra, India). Gram-stained cells were observed under a light microscope (CX40, China) at 100× magnification; images were captured with a SOPTOP OD400UHW-P digital microscope camera.

Phase-contrast microscopy was used to observe bacterial cell morphology without staining. The bacterial strain pre-grown on YEG plates was collected in 5 mL of 0.9% NaCl using sterile cotton swabs. Ten µL of the suspension was placed on a glass slide, and observations were performed with a phase-contrast microscope (Zeiss Primostar 3) at 100× magnification; images were recorded at the selected magnification using a digital imaging system (Axiocam 212 color microscope camera).

For Scanning Electron Microscopy (SEM) and Transmission Electron Microscopy (TEM), the bacterial strain JSM-10 was grown in liquid YEG medium for 24 h at 130 rpm, with an initial density of 1 × 10^5^ CFU/mL. After incubation, 1 mL of the culture was transferred to a 2 mL Eppendorf tube containing 1 mL of 2.5% glutaraldehyde (GA), and then it was mixed gently. The prepared sample was stored at 4 °C overnight for fixation prior to submission for SEM and TEM analyses to an external service (Electron Microscopy Laboratory, Core Facilities and HPC, Nazarbayev University).

For SEM analysis (Auriga Crossbeam 540, Carl Zeiss, Oberkochen, Germany), bacterial cells were collected by centrifugation and fixed in 2.5% GA prepared in phosphate buffer (pH 7.2–7.4) at 4 °C for 1–2 h. The samples were then washed with the same buffer and post-fixed in 1% osmium tetroxide for approximately 1 h. Subsequently, the samples were dehydrated through a graded ethanol series (30–100%). The dehydrated specimens were placed onto collagen-coated coverslips. After drying, the samples were mounted on holders and coated with a thin conductive carbon layer prior to imaging.

For TEM analysis (JEM 1400 Plus, JEOL, Tokyo, Japan), cells were fixed in 2.5% GA in phosphate buffer (pH 7.2–7.4) at 4 °C for 1–2 h, followed by washing in buffer and post-fixation in 1% osmium tetroxide for approximately 1 h. The samples were then dehydrated in a graded ethanol series (30–100%), infiltrated with epoxy resin, and polymerized at 60 °C. Ultrathin sections (60–90 nm) were prepared from the polymerized blocks using an ultramicrotome (Leica UC7, Wetzlar, Germany).

### 4.2. Abiotic Stress Tolerance of JSM-10

#### 4.2.1. Glyphosate Tolerance Evaluation

Glyphosate tolerance of the isolated JSM-10 strain was examined using a broth microdilution method. The YEG medium supplemented with glyphosate “Uragan Forte” (500 g/L) at final concentrations of 0.05, 0.1, 0.2, 0.5, 1, 2, and 5 g/L was dispensed (180 µL) to a 96-well Nunc microplate (ThermoFisher, Rochester, NY, USA). Inoculated YEG medium without glyphosate and non-inoculated YEG containing corresponding glyphosate concentrations served as the control and blank samples, respectively. Bacterial cell suspension was collected from overnight-grown YEG plate cultures in 5 mL 0.9% NaCl using cotton swabs, and the cell density was measured at OD_620_. Based on the measured cell density, the subsequent bacterial suspension for inoculation was adjusted to 1 × 10^6^ CFU/mL in 5 mL 0.9% NaCl. The bacterial suspension (20 µL) was used to inoculate each well containing 180 µL YEG with or without glyphosate, resulting in an initial density of 1 × 10^5^ CFU/mL. The plate was incubated in a temperature-controlled CAPP Rondo 4-Place Incubating Shaker, CRPI-412X (AHN Biotechnologie GmbH, Nordhausen, Germany) at 25 °C and 200 rpm. Cell density was measured at OD_620_ after 24 and 48 h of incubation using a Multiskan SkyHigh Microplate Spectrophotometer (Thermo Fisher Scientific, Singapore). The described protocol for bacterial suspension preparation, inoculation, and incubation was applied throughout this subsection in order to ensure quantifiability, reproducibility, and compatibility among all tests. Measurements at 24 and 48 h were consistently applied in all subsequent stress tolerance assays to evaluate the time-dependent adaptation of the strain to adverse environmental conditions. All analyses, unless specified, were performed in triplicate.

#### 4.2.2. Examination of Salinity Tolerance

For testing salinity tolerance, the bacterial strain was incubated in a 96-well microplate containing 180 µL liquid YEG adjusted with different concentrations of NaCl (g/L: 0, 15, 20, 25, 30, 35, 40, and 45), where YEG without NaCl served as the control. Cell suspension preparation, inoculation, incubation, and growth monitoring (at 24 and 48 h) were performed according to the protocols described in Section 4.2.1.

#### 4.2.3. Drought Tolerance Assay

Polyethylene glycol with a molecular mass of 6000 (PEG 6000) was used to simulate osmotic stress conditions [68]. The strain was cultured in YEG supplemented with PEG 6000 at final concentrations of 0 (applied as the control), 25, 50, 100, 150, 200, 250, and 300 g/L. Inoculation, incubation conditions, and growth monitoring (OD_620_ at 24 and 48 h) were conducted as detailed in Section 4.2.1.

#### 4.2.4. Determination of an Optimal Temperature Range

The temperature range (10, 15, 20, 25, 30, 35, 40, and 45 °C) suitable for growth of *Paenibacillus* sp. JSM-10 was evaluated in YEG medium using the standardized inoculation and growth measurement protocols described in Section 4.2.1.

#### 4.2.5. Heat-Shock Stress Examination

The heat-shock method was applied to study the resilience of JSM-10 to sharp temperature fluctuations occurring under field conditions. Heat-shock tolerance was mimicked by exposing 1 mL of cell suspension (prepared in 0.9% NaCl at 1 × 10^7^ CFU/mL in 2 mL safe-lock Eppendorf tubes) to a range of temperatures of 30, 40, and 50 °C as mild, moderate, and severe shock levels; and for three exposure times: short, medium, and long of 5, 30, and 60 min, respectively. After heat exposure, 100 µL of each treated sample was transferred into wells of a 96-well plate containing 100 µL of fresh sterile double-concentrated YEG medium. As the control, YEG was inoculated with 100 µL of cell suspension maintained at 25 °C throughout the heat-shock assay. Bacterial recovery was measured after 24 h of incubation at 25 °C with shaking at 200 rpm in a shaker-incubator using a Multiskan SkyHigh microplate spectrophotometer at OD_620_. The assay was run in three replicates.

### 4.3. Plant Growth-Promoting Traits of JSM-10 Strain

#### 4.3.1. Indole-3-Acetic Acid (IAA) Synthesis

IAA production was evaluated using a rapid plate assay with minor modifications [69]. Bacterial cell suspensions were prepared in 5 mL 0.9% NaCl at final concentrations of 1 × 10^5^, 1 × 10^6^, and 1 × 10^7^ CFU/mL. An aliquot of 200 μL from each cell suspension was transferred into 1 cm diameter holes made in YEG agar supplemented with 0.1 g/L tryptophan as a precursor of IAA synthesis. Holes containing 200 μL sterile 0.9% NaCl served as the non-inoculated control. Following a five-day incubation at 25 °C, the holes were cleared of bacterial biomass, and 200 μL of Salkowski reagent (12 g/L FeCl_3_ in 37% H_2_SO_4_) [70] was added. The reaction was incubated for 20 min at 25 °C in the dark, and IAA production was indicated by the development of a pink-yellow coloration surrounding the holes. All assays were performed in triplicate.

IAA concentration in bacterial culture supernatants was determined using a calibration curve. Strain JSM-10 was cultivated in YEG supplemented with 0.1 g/L L-tryptophan, and the supernatant was collected on the third day after incubation at 25 °C and 130 rpm. For color development, 100 µL of sample or standard solution was mixed with 100 µL of Salkowski reagent and incubated for 20 min at 25 °C in the dark. Absorbance was measured at 530 nm. The exact IAA concentrations in the supernatants were determined using the constructed calibration curve in SkanIT software (version 7.0.2). For the calibration curve, a 0.5 mg/mL IAA stock solution was prepared in DMSO and used to obtain standards ranging from 0 to 500 µg/mL. Standards were treated in the same way as the bacterial supernatant samples and measured in triplicate. A calibration curve was constructed using linear regression and used to determine IAA concentrations in bacterial culture supernatants.

#### 4.3.2. Phosphorus Solubilization Capacity

Phosphate solubilization was evaluated using Pikovskaya agar (g/L: glucose 10; (NH_4_)_2_SO_4_ 0.5; MgSO_4_·7H_2_O 0.1; KCl 0.2; yeast extract 0.5; NaCl 0.2; MnSO_4_·H_2_O 0.002; FeSO_4_·7H_2_O 0.002; agar 20) [71] supplemented with 5 g/L CaHPO_4_ as the inorganic phosphate source [72]. Bacterial suspensions were adjusted to 1 × 10^5^, 1 × 10^6^, and 1 × 10^7^ CFU/mL, and 10 µL aliquots were spot-inoculated onto the agar plates in triplicate and air-dried for 10 min. After 10 days of incubation at 25 °C, well-defined halo zones surrounding the bacterial colonies were observed, indicating the phosphorus solubilization potential.

Colony diameter and halo-zone diameter were measured, and the phosphorus solubilization index was calculated according to the following formula:

Solubilization index = (Diameter of solubilization zone, mm + Diameter of colony growth, mm)/(Diameter of colony growth, mm)(1)

#### 4.3.3. Siderophore Production

The siderophore-producing capacity of the JSM-10 isolate was investigated using the Chrome Azurol S (CAS) agar method [73]. The CAS reagent was prepared in distilled water by mixing 10 mL of 1 mM FeCl_3_·6H_2_O in 10 mM HCl with 50 mL of 2 mM CAS solution, followed by the addition of 40 mL of 5 mM hexadecyltrimethylammonium bromide (HDTMA). Bacterial suspensions at target concentrations were prepared as described above, and 10 µL aliquots of each suspension were inoculated onto LB agar (g/L: peptone 7.5; NaCl 2.5; yeast extract 2.5; agar 15), mixed with autoclaved CAS reagent at a 1:1 ratio [74], air-dried for 10 min, and incubated at 25 °C for 7 days. Siderophore production was indicated by the formation of an orange halo zone around the colonies. All assays were tested in three replicates.

#### 4.3.4. The Effect of JSM-10 and Its Culture Filtrates on Buckwheat (*Fagopyrum esculentum* L.) Growth with and Without Saline Conditions

The experiment was designed to assess both the direct effect of living cultures of *Paenibacillus* sp. JSM-10 and the contribution of its extracellular metabolites (CCF) on buckwheat growth with and without exposure to saline stress conditions. Plastic pots (25 mL) were filled with 10 g of soil substrate. To simulate salinity stress, 3 mL of 125 mM NaCl solution was added to the substrate and allowed to dry for 24 h. For non-saline conditions, 3 mL of distilled water (dH_2_O) was applied and dried under the same conditions. At the time of sowing, three treatments were applied. The JSM-10 treatment consisted of 2 mL of a 3-day culture of strain JSM-10 grown in Minimal Medium supplemented with glucose (MM + Glu), combined with 1 mL of the corresponding cell-free culture filtrate (CCF) (obtained as described below in Section 4.4.2) and 2 mL dH_2_O. The medium control (MC-I), included to control for medium effects, received 3 mL of CCF from non-inoculated MM + Glu and 2 mL of dH_2_O, while the water control (WC-II) received 5 mL of dH_2_O. Seven seeds were sown in each pot and covered with approximately 5 g of soil.

On the third day of incubation, a secondary treatment was applied. For the JSM-10 treatment, 2 mL of a 24 h culture grown in YEG supplemented with 0.5 g/L tryptophan was combined with 1 mL of CCF obtained from JSM-10 grown in MM + Glu and 2 mL of dH_2_O. The medium control received 2 mL of non-inoculated YEG supplemented with 0.5 g/L tryptophan, 1 mL of CCF from non-inoculated MM + Glu, and 2 mL of H_2_O. The water control received 5 mL of dH_2_O.

Plant growth parameters were assessed on the fifth day after sowing. Root length, shoot (seedling) length, and total seedling length (sum of root and shoot) were recorded. All treatments were performed in triplicate and incubated at 25 °C under a natural day/night cycle.

### 4.4. Antagonistic Activity Assays

#### 4.4.1. Inhibition of Fungal Plant Pathogens

Initial assessment of the antagonistic potential of bacterial strain JSM-10 was evaluated against two phytopathogenic fungal isolates (*Bipolaris sorokiniana* W-100 and *Alternaria* spp. W-150) isolated in this study, following the method described by [75]. Once the antagonistic potential was confirmed, additional strains (*Alternaria* spp. 4/1, 8/7, 11/1, 41/1, 42/1, and *Nigrospora oryzae* 22/1) were included in the study. Identification of the newly isolated strains *B. sorokiniana* W-100 and *Alternaria* spp. W-150 (submitted in NCBI under accession number: PZ067503 and PZ067504) was performed as reported for the retrieved fungal strains [76]. The fungal isolates were maintained on YEG and incubated at 25 °C for 3–7 days, depending on growth rate. For the antagonism assay, a 6 mm diameter agar plug containing actively growing mycelium was transferred to the center of a fresh YEG plate. To evaluate the inhibitory capacity of JSM-10 under different bacterial inoculum loads, two co-cultivation designs were applied. In the high-inoculum treatment, a 24 h culture of strain JSM-10 was streaked using sterile cotton swabs at a distance of 2.5 mm from the fungal plug on four equidistant sides. In the low-inoculum treatment, the bacterial strain was spot-inoculated using sterile toothpicks at a distance of 3.5 mm from the fungal plug. The control plates consisted of fungal isolates grown without bacterial inoculation. The experiment was carried out in triplicate. Plates were incubated at 25 °C for 7 days. The inhibitory activity (IR) was determined using the following formula:

IR = (D1 − D2)/D1 × 100%(2)

where IR represents the percentage of inhibitory activity, D1 is the diameter of the phytopathogen colony in the control (mm), and D2 is the diameter of the phytopathogen colony (mm) in the presence of the JSM-10 strain [77].

#### 4.4.2. Antagonistic Activity of Living Cultures of *Paenibacillus* sp. JSM-10 and Its Culture Filtrates Towards *Escherichia coli*

The antagonistic activity of the JSM-10 strain against *E. coli* DH5α was evaluated using a co-culture assay. Cell suspensions of *Paenibacillus* sp. JSM-10 were prepared at final concentrations of 1 × 10^5^, 1 × 10^6^, and 1 × 10^7^ CFU/mL (to apply low-, moderate-, and high-concentration treatment, respectively) while *E. coli* was prepared at 1 × 10^5^ CFU/mL. Then, 5 mL of the prepared *E. coli* suspension was used to cover the surface of YEG agar plates, and the excess was removed. The inoculated plates were air-dried for 15 min, followed by spot inoculation of 10 µL of each bacterial suspension (three concentrations per plate). The plates were air-dried for an additional 10 min and then incubated at 25 °C for 5 days. Individual plates inoculated solely with *E. coli* or JSM-10 served as the untreated controls. Antagonistic activity was assessed based on the presence of visible inhibition zones surrounding JSM-10 spots, presented in Supplemental Figure S3.

For obtaining the cell-free culture filtrate (CCF) to evaluate potential inhibitory metabolites, JSM-10 was inoculated at 1 × 10^5^ CFU/mL as the initial cell density in 50 mL Minimal Medium (g/L: KH_2_PO_4_, 1.0; MgSO_4_·7H_2_O, 0.5; (NH_4_)_2_SO_4_, 5.0) in distilled water [78], supplemented individually with 2 g/L glucose or L-alanine. Three-day shaking cultures grown at 25 °C in an orbital shaker (IKA KS 260, Staufen im Breisgau, Germany) at 130 rpm were centrifuged at 10,000× *g* for 10 min, and the collected supernatant was filter-sterilized via 0.22 µm syringe filters (TPP, Schaffhausen, Switzerland). The sterility of the obtained CCF was confirmed by plating 10 µL on YEG agar, followed by incubation for 3 days at 25 °C with daily monitoring for growth. The CCF of non-inoculated MM prepared in the same way served as the control (C-CCF) to evaluate the medium effects on *E. coli* growth. The CCF was stored at 4 °C during the experimental period.

The antagonistic activity of CCF produced by strain JSM-10 against *E. coli* was evaluated using a microplate-based kinetic growth assay. Liquid YEG was amended with the obtained CCF at final concentrations of 25 and 50% (*v*/*v*), while YEG without CCF was set as the untreated control. Aliquots of 10 µL of *E. coli* suspension adjusted to 1 × 10^6^ CFU/mL were added to wells containing 90 µL of YEG medium with or without CCF, resulting in an initial cell density of 1 × 10^5^ CFU/mL per well. All treatments were performed in triplicate. The 96-well plate was incubated at 37 °C in a Multiskan SkyHigh microplate spectrophotometer operating in kinetic mode. Bacterial growth was monitored by measuring absorbance at OD_620_ at 20 min intervals for 24 h without shaking. The inhibition rate (%) was derived from final-cycle OD_620_ measurements and expressed as the relative decrease in *E. coli* growth in CCF-treated samples compared to the untreated control.

### 4.5. Statistical Analyses and Data Visualization

Statistical analyses were performed using one-way analysis of variance (ANOVA) to evaluate the effects of abiotic stress factors (glyphosate, salinity, and drought) on bacterial growth. Prior to ANOVA, all data were tested for normality using the Shapiro–Wilk test and for homogeneity of variances using Levene’s test from the *car* package [79]. For variants that did not meet the assumptions for parametric ANOVA, a non-parametric alternative (Kruskal–Wallis test) was applied. The significance threshold was applied as 95% (*p* < 0.05). When ANOVA indicated significant differences, the data were subsequently analyzed using Tukey’s honestly significant difference (HSD) post hoc comparisons from the *agricolae* package [80]. The significance threshold was set at 95% (*p* < 0.05).

Temperature range and heat-shock experiments were conducted as physiological tolerance assays to determine growth and survival ranges. Therefore, the results were evaluated descriptively (means and standard deviations) based on growth patterns and recovery trends rather than inferential statistical comparisons.

In the plant growth assessment studies, two-way ANOVA was performed to evaluate the effects of bacterial treatment (JSM-10 inoculation vs. controls) and NaCl exposure on buckwheat root length. The assumptions of ANOVA were tested prior to analysis, as described above. As no significant interaction between factors was detected in the full factorial model, the additive model was applied. Tukey’s HSD test was used for post hoc pairwise comparisons. In addition, to quantify the magnitude of the difference between the JSM-10 strain and the respective controls (MC-I and WC-II), effect sizes were calculated using Hedges’ *g* via the *effectsize* package [81]. Hedges’ *g* was selected over Cohen’s *d* to provide a more accurate estimate for *n* = 3 sample sizes, employing a pooled standard deviation. Hedges’ *g* values were calculated separately for each NaCl condition. All effect sizes are reported with their corresponding 95% confidence intervals (CI). Descriptive statistics (means and standard deviations) were calculated for each experimental group (*n* = 3 per group) and presented in Figure 6.

All graphs were constructed using the *ggplot2* package [82] and merged using the *patchwork* package [83]. All statistical analyses and data visualization were conducted in the R statistical environment (R Core Team, version 4.4.1; https://www.r-project.org/contributors.html (accessed on 26 April 2026)) using RStudio, version 2023.06.1 (Posit, https://posit.co/download/rstudio-desktop (accessed on 26 April 2026)). All data are presented as mean ± standard deviation of three biological replicates, unless otherwise stated.

## 5. Conclusions

This study expands current knowledge by providing an integrative assessment of the *Paenibacillus* sp. JSM-10 strain, including its detailed microscopic characterization, tolerance to multiple abiotic stresses, resistance to glyphosate, time-dependent responses to salinity and osmotic stress, plant growth-promoting trend, and antagonistic activity. The observed functional diversity suggests the involvement of multiple yet insufficiently explored molecular mechanisms, potentially including phytohormone-mediated signaling, stress-responsive metabolic pathways, and metabolite-driven antagonistic interactions.

Importantly, the demonstrated tolerance to environmentally relevant stress factors, together with the multifunctional plant-beneficial traits, supports the potential applicability of glyphosate-tolerant *Paenibacillus* sp. JSM-10 as a functional, stress-resilient PGPB candidate for improving crop performance under abiotic stress in sustainable agricultural systems.

The present study represents the foundational stage in the development and evaluation of a novel plant growth-promoting bacterial inoculant. Ongoing and future research, including genomic and molecular analyses, ecological safety assessments, greenhouse experiments, and multi-site field validation, will be essential to further evaluate the environmental safety and agricultural efficiency of strain JSM-10. These subsequent studies will support the further development of *Paenibacillus* sp. JSM-10 as a promising stress-tolerant microbial inoculant for sustainable agriculture.

## Figures and Tables

**Table 1:** Antagonistic potential of *Paenibacillus* sp. JSM-10 against different pathogenic species.

Sample	CD, cm /OD_620_	Inhibition Rate, %
**Fungal Plant Pathogens, Co-Culture Plate Assay**		
***Bipolaris sorokiniana* W-100, C**	3.98 ± 2.14	-
*B. sorokiniana* W-100 + JSM-10, I	2.00 ± 0.18	49.7
*B. sorokiniana* W-100 + JSM-10, II	1.43 ± 0.19	64.2
***Nigrospora oryzae* 22/1, C**	8.10 ± 0.17	-
*N. oryzae* 22/1 + JSM-10, I	2.43 ± 0.12	70.0
*N. oryzae* 22/1 + JSM-10, II	2.33 ± 0.15	71.2
***Alternaria* spp. W-150, C**	7.03 ± 0.29	-
*Alternaria* spp. W-150 + JSM-10, I	2.33 ± 0.30	66.9
*Alternaria* spp. W-150 + JSM-10, II	3.25 ± 0.33	53.7
***Alternaria* spp. 4/1, C**	5.73 ± 1.37	-
*Alternaria* spp. 4/1 + JSM-10, I	2.03 ± 0.31	64.5
*Alternaria* spp. 4/1 + JSM-10, II	2.66 ± 0.21	53.5
***Alternaria* spp. 8/7, C**	3.85 ± 0.07	-
*Alternaria* spp. 8/7 + JSM-10, I	1.63 ± 0.23	57.6
*Alternaria* spp. 8/7 + JSM-10, II	2.43 ± 0.15	36.8
***Alternaria* spp. 11/1, C**	6.60 ± 1.14	-
*Alternaria* spp. 11/1 + JSM-10, I	2.16 ± 0.23	67.2
*Alternaria* spp. 11/1 + JSM-10, II	2.96 ± 0.31	55.1
***Alternaria* spp. 41/1, C**	5.65 ± 0.64	-
*Alternaria* spp. 41/1 + JSM-10, I	1.76 ± 0.45	68.7
*Alternaria* spp. 41/1 + JSM-10, II	2.26 ± 0.06	59.9
***Alternaria* spp. 42/1, C**	6.33 ± 1.53	-
*Alternaria* spp. 42/1 + JSM-10, I	1.76 ± 0.15	72.1
*Alternaria* spp. 42/1 + JSM-10, II	2.70 ± 0.10	57.4
***Escherichia coli*, kinetic measurements**		
***E. coli*** **, C**	0.15 ± 0.001	-
JSM-10, Ala, 50%	0.08 ± 0.001	45.1
C-CCF, Ala, 50%	0.13 ± 0.012	12.6
JSM-10, Ala, 25%	0.14 ± 0.005	6.8
C-CCF, Ala, 25%	0.14 ± 0.002	5.1
JSM-10, Glu, 50%	0.08 ± 0.001	45.4
C-CCF, Glu, 50%	0.14 ± 0.008	6.8
JSM-10, Glu, 25%	0.14 ± 0.005	4.1
C-CCF, Glu, 25%	0.14 ± 0.013	7.8

**For fungi, co-culture plate assay:** C is the control (growth in the absence of JSM-10), highlighted in bold for convenience, while bacterial-treated plates are indicated as “+JSM-10”; I and II represent high- and low-inoculum treatments, respectively. **For *E. coli*, kinetic assay:** C is the untreated control (growth in the absence of CCF), highlighted in bold; C-CCF is the control treated with CCF (growth in the presence of filtrates from non-inoculated MM); JSM-10 denotes treatment with CCF obtained from JSM-10 (growth in the presence of CCF of JSM-10 grown in MM); Ala and Glu are filtrates from MM amended with L-alanine or glucose, respectively; 25 and 50% represent the applied CCF concentrations.

**Table 2:** List of reported PGPB of *Paenibacillus* species with beneficial effects on different crops.

*Paenibacillus* Species	Tested Crops/Pest	Reported Effect	References
**PGPB Characteristics**			
*Paenibacillus nicotianae* AFI2	Wheat (*Triticum aestivum* L.)	Increased shoot length by 21.1% under Ni stress	[23]
*Paenibacillus peoriae* MHJL1	Cotton (*Gossypium hirsutum*)	Plant growth; increased plant height, root length, stem diameter and fresh weight by 14.12–120.47%	[17]
*Paenibacillus beijingensis* BJ-18	Wheat (*T. aestivum* L.)	Increased shoot and root dry weight by 86.1% and 46.0% under low-nitrogen conditions	[24]
	Maize (*Zea mays*)	Increased shoot and root dry weight by 46.6% and 47.5% under low-nitrogen conditions	
	Cucumber (*Cucumis sativus*)	Increased shoot and root dry weight by 103.6% and 20.3% under low-nitrogen conditions	
*Paenibacillus polymyxa* ZYPP18	Wheat (*T. aestivum* L.)	Enhanced seedling growth and reduced disease incidence by 37.4–65.6%	[25]
*Paenibacillus* sp.	Wheat (*T. aestivum* L.)	Increased shoot length by 30.9%	[26]
	Cucumber (*C. sativus*)	Increased shoot and root length by 50.0% and 94.4%	
	Tomato (*Solanum lycopersicum*)	Increased shoot and root length by 64.6% and 55.2%	
*P. polymyxa* 92	Wheat (*T. aestivum* L.)	Increased shoot and root length up to 22% and dry weight up to 28%	[27]
*Paenibacillus mucilaginosus* G78	Tomato (*S. lycopersicum*)	Increased plant height and fresh weight by 44.1% and 90.0%	[28]
*Paenibacillus illinoisensis* YZ29	Peanut (*Arachis hypogaea*)	Increased yield by 37.05%	[29]
**Antagonistic activity**			
*P. peoriae* GXUN15128	in vitro (plant-pathogenic fungi)	Inhibited growth of 10 fungal species by 48.4–86.1% (in vitro)	[20]
*P. peoriae* 3-B4	*Fusarium verticillioides*	Inhibited growth by 59.92%	[30]
*P. polymyxa* ZYPP18	*Rhizoctonia cerealis*	Inhibited fungal growth by 92.68%	[25]
*Paenibacillus* sp.	*Fusarium graminearum*	Formed inhibition zones larger than 25 mm	[26]
	*Fusarium solani*	Formed inhibition zones of 5–15 mm	
*Paneibacillus tianmuensis* YM002	*Acidovorax citrulli* (cucumber leaves)	Formed inhibition zones with diameters ranging from 1.95 to 9.97 mm	[31]
*P. polymyxa* AF01	*Botrytis cinerea*	Inhibited growth by 78.29%	[32]
	*Bipolaris cactivora*	Inhibited growth by 60.94%	
	*Fusarium equiseti*	Inhibited growth by 66.28%	
*P. polymyxa* SK1	*Botryosphaeria dothidea*	Inhibited growth by 66.67%	[33]
	*B. cinerea*	Inhibited growth by 61.19%	
	*Fusarium fujikuroi*	Inhibited growth by 60.71%	
	*Fusarium oxysporum*	Inhibited growth by 55.54%	
*Paenibacillus jamilae* HS-26	*F. oxysporum*	Inhibited growth by 46.30%	[34]
	*Bipolaris sorokiniana*	Inhibited growth by 63.86%	
	*Rhizoctonia solani*	Inhibited growth by 44%	

**Table 3:** Molecular detection of functional genes in JSM-10.

Gene	Amplicon Size (bp)	Function	Reference
**PGP Characteristics**			
*gcd*	601	Glucose-1-dehydrogenase; involved in oxidation of glucose to gluconic acid	[61]
*ipdC*	951	Indole-3-pyruvate decarboxylase; involved in indole-3-acetic acid (IAA) biosynthesis	[42,61]
**Abiotic stress tolerance**			
*thiO*	815	Glycine oxidase; involved in glyphosate degradation	[62]
*ectA*	740	Diaminobutyric acid acetyltransferase; involved in ectoine biosynthesis (osmoprotection)	[63]
*groL*	575	Chaperonin GroEL; assists in protein folding under normal and stress conditions	[63]

**Table 4:** Primer design and PCR programs for differential genes of JSM-10.

Amplified Genes and Corresponding Primers F: 5′-3′ R: 3′-5′	PCR Program	References
**16S rRNA** Eub 341-F: CCTACGGGAGGCAGCAG Eub 1060-R: CGACACGAGCTGACGACA	95 °C, 2 min (1 cycle); 95 °C, 30 s; 57 °C, 45 s; 72 °C, 1 min (35 cycles); 72 °C, 7 min (1 cycle)	[66]
***gyrA* fragment gyrA1** FW_434_gyrA_1: GCATTAACCTCTTGCTCCTTGAAGCGTAT RV_1326_gyrA_1: TGGAAGGTTTGGTCAAGGCGCTGAACATTC	95 °C, 30 s (1 cycle); 95 °C, 15 s; 58 °C, 20 s; 72 °C, 58 s (30 cycles), 72 °C, 5 min (1 cycle)	This study
***gyrA* fragment gyrA2** FW_1775_gyrA_2: TCCTTGATGCCTTCGCCACCCATAATGAGTC RV_2415_gyrA_2: GTCGGTGACCGCTTTGGCCGATATTCCA	95 °C, 30 s (1 cycle); 95 °C, 15 s; 62 °C, 20 s; 72 °C, 58 s (30 cycles), 72 °C, 5 min (1 cycle)	This study
***rho*** FW_rho: GCCAATAGCATTTCTACCAACAATCCCG RV_rho: GTTGTTGCCCACCAGAACTGCTTTG	95 °C, 30 s (1 cycle) 95 °C, 15 s; 56 °C, 20 s; 72 °C, 40 s (32 cycles) 72 °C, 2 min (1 cycle)	This study
***gcd*** FW_gcd: CCCAATGTAAAGAAGTTCCGATTGC RV_gcd: CTGACAATGGCTCCTTTGGTAGCTG	95 °C, 30 s (1 cycle) 95 °C, 15 s; 63 °C, 20 s; 72 °C, 40 s (32 cycles) 72 °C, 2 min (1 cycle)	This study
***ipdC*** FW_ipdC: CCTGAAGTTAGGTCAACCAATGAATTAC RV_ipdC: CAATGTGGACGATTTTAGCTTGAGGAG	95 °C, 30 s (1 cycle) 95 °C, 15 s; 54 °C, 20 s; 72 °C, 57 s (32 cycles) 72 °C, 2 min (1 cycle)	This study
***thiO*** FW_thiO: CTGAATGCTTGGTCATAGGTGGAGGTG RV_thiO: CTACGGTCACTTCTTCGTCATATTGATG	95 °C, 30 s (1 cycle) 95 °C, 15 s; 53 °C, 20 s; 72 °C, 57 s (32 cycles) 72 °C, 2 min (1 cycle)	This study
***ectA*** FW_ectA: CTGTTAAAGTAACACTCGGACCGAAAG RV_ectA: CAGCGTTGAATGTACCACGCAGTTTG	98 °C, 3 min (1 cycle) 98 °C, 15 s; 69 °C, 20 s; 72 °C, 18 s (31 cycles) 72 °C, 2 min (1 cycle)	This study
***groL*** FW_groL: CGAACGATGTAGCTGGTGATGGTAC RV_groL: CAGCGTTGAATGTACCACGCAGTTTG		This study

## Data Availability

The sequences of 16S rRNA gyrA2, gyrA1 of *gyrA* and *rho* of *Paenibacillus* sp. JSM-10 and ITS of fungal isolates obtained during this data were uploaded to NCBI (accession numbers PZ059921, PZ241072, PZ287672, PZ316596, PZ067503, and PZ067504, respectively). All collected data of this study are available on a reasonable request from the corresponding author.

## Supplementary Materials

The following supporting information can be downloaded at: https://www.mdpi.com/article/10.3390/ijms27094062/s1.
