# Supplementary material for: Integrative Exploration of Paenibacillus sp. JSM-10 as a Potential Multi-Stress-Tolerant Microbial Inoculant for Sustainable Agriculture
Source: Int J Mol Sci. 2026 Apr 30;27(9):4062. doi: 10.3390/ijms27094062 (PMC13164025; doi:10.3390/ijms27094062)
Supplement: Supplementary file 1 [file ijms-27-04062-s001.zip › ijms-4202651-supplementary.pdf]

Supplementary Figures

Phylogenetic analysis

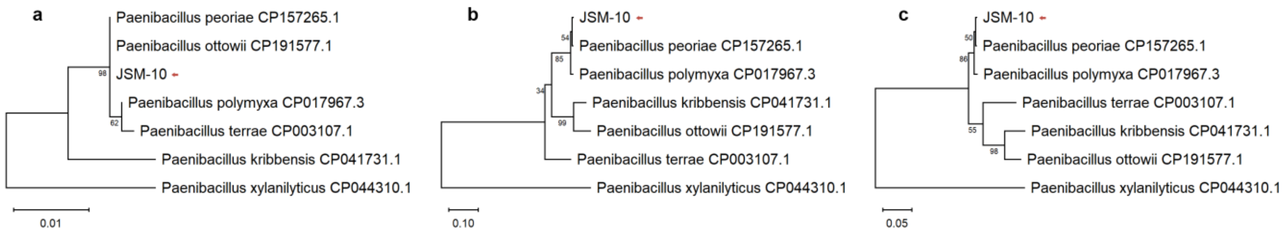

Figure S1. Phylogenetic analysis of strain JSM-10 and closely related *Paenibacillus* species based on different genetic markers. (**a**) Maximum Likelihood phylogenetic tree based on 16S rRNA gene sequences. (**b**) Maximum Likelihood phylogenetic tree based on *gyrA* partial gene sequence (gyrA1 fragment) sequences. (**c**) Maximum Likelihood phylogenetic tree based on concatenated 16S rRNA and *gyrA* gene (gyrA1 fragment) sequences. Bootstrap values (1000 replicates) are shown at branch nodes. The strain JSM-10 is indicated with a red arrow.

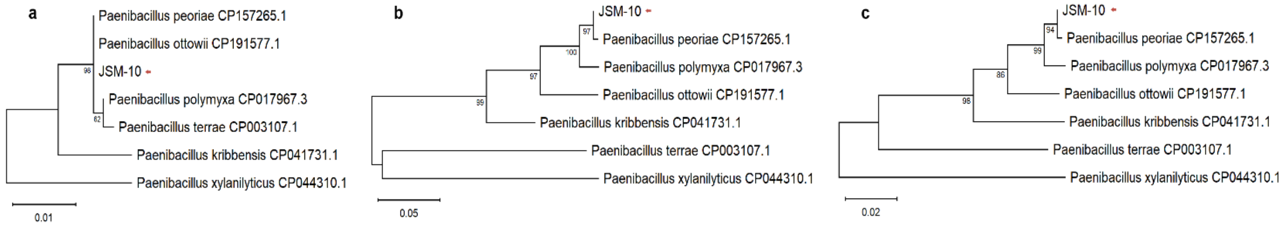

Figure S2. Phylogenetic analysis of strain JSM-10 and closely related *Paenibacillus* species based on different genetic markers. (**a**) Maximum Likelihood phylogenetic tree based on 16S rRNA gene sequences. (**b**) Maximum Likelihood phylogenetic tree based on *rho* partial gene sequence. (**c**) Maximum Likelihood phylogenetic tree based on concatenated 16S rRNA and *rho* gene sequences. Bootstrap values (1000 replicates) are shown at branch nodes. The strain JSM-10 is indicated with a red arrow.

Antagonistic activity against *E. coli* DH5α

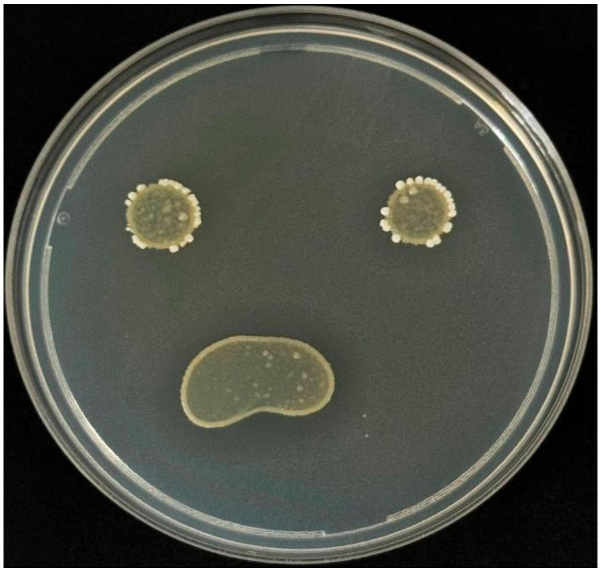

Figure S3. Antibacterial activity of strain JSM-10 against *Escherichia coli* (1×10^5 CFU/mL), evaluated by agar plate assay. The plate shows bacterial colony formation and inhibition pattern after incubation, indicating antagonistic activity of strain JSM-10 against *E. coli*.

Supplementary Figures of Scanning Electron Microscope (SEM) and Transmission Electron Microscope (TEM) images showing cellular morphology of *Paenibacillus* sp. JSM-10 at different magnifications. Scale bars are indicated in each panel.

SEM:

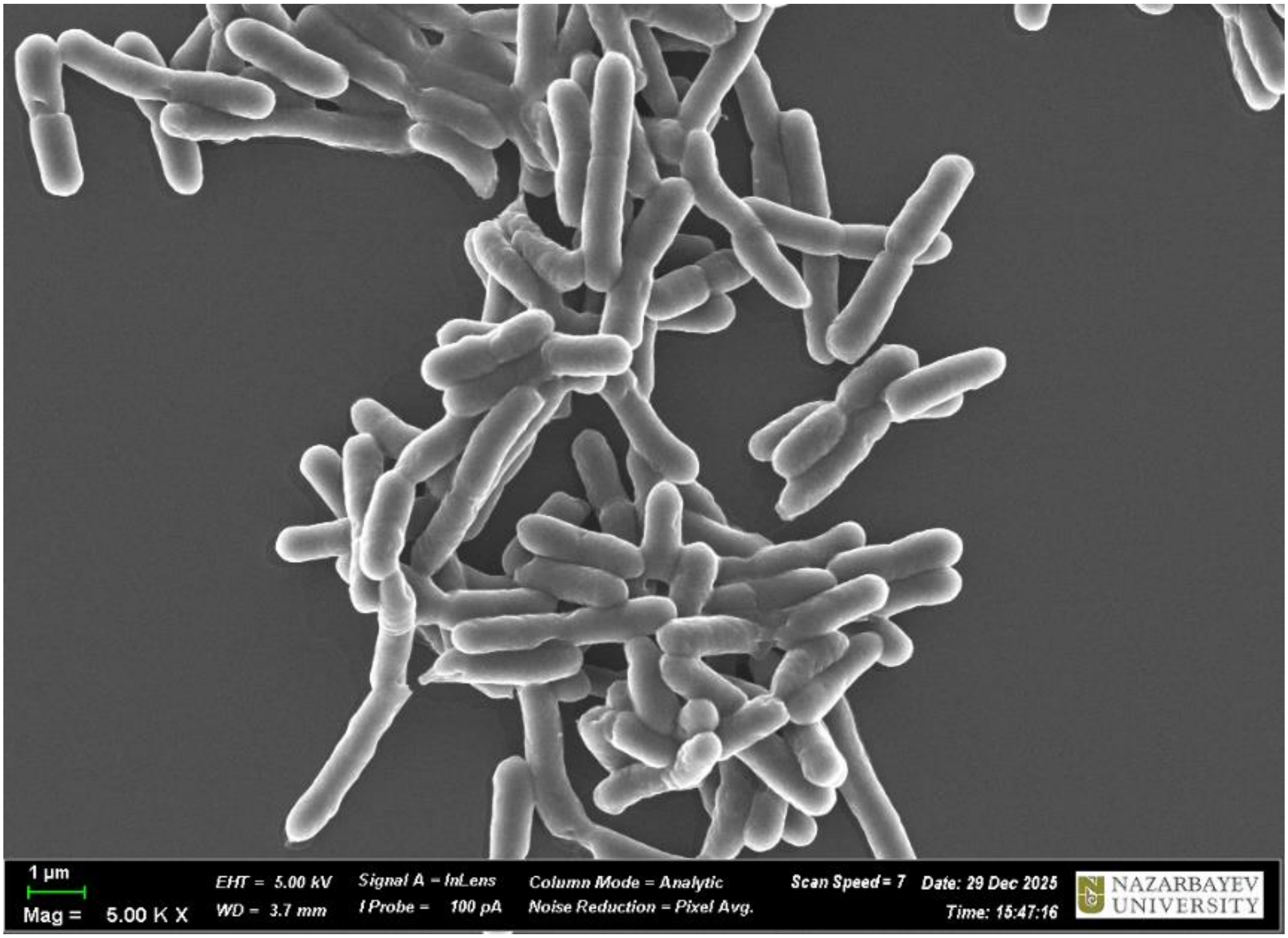

Figure S4. SEM of strain JSM-10 as shown in **Figure 2d (uncropped original image).**

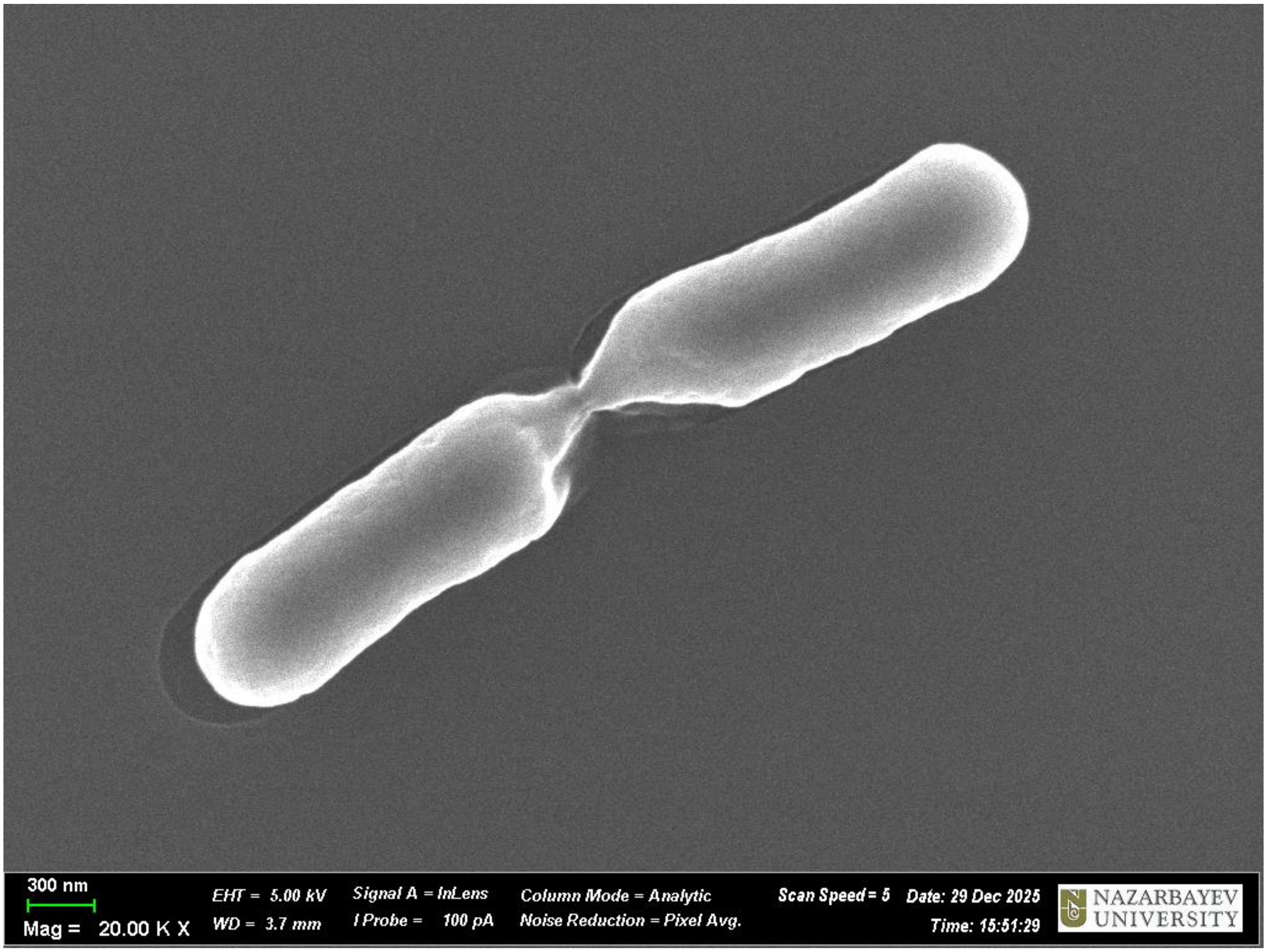

Figure S5. SEM of strain JSM-10 as shown in **Figure 2e (uncropped original image).**

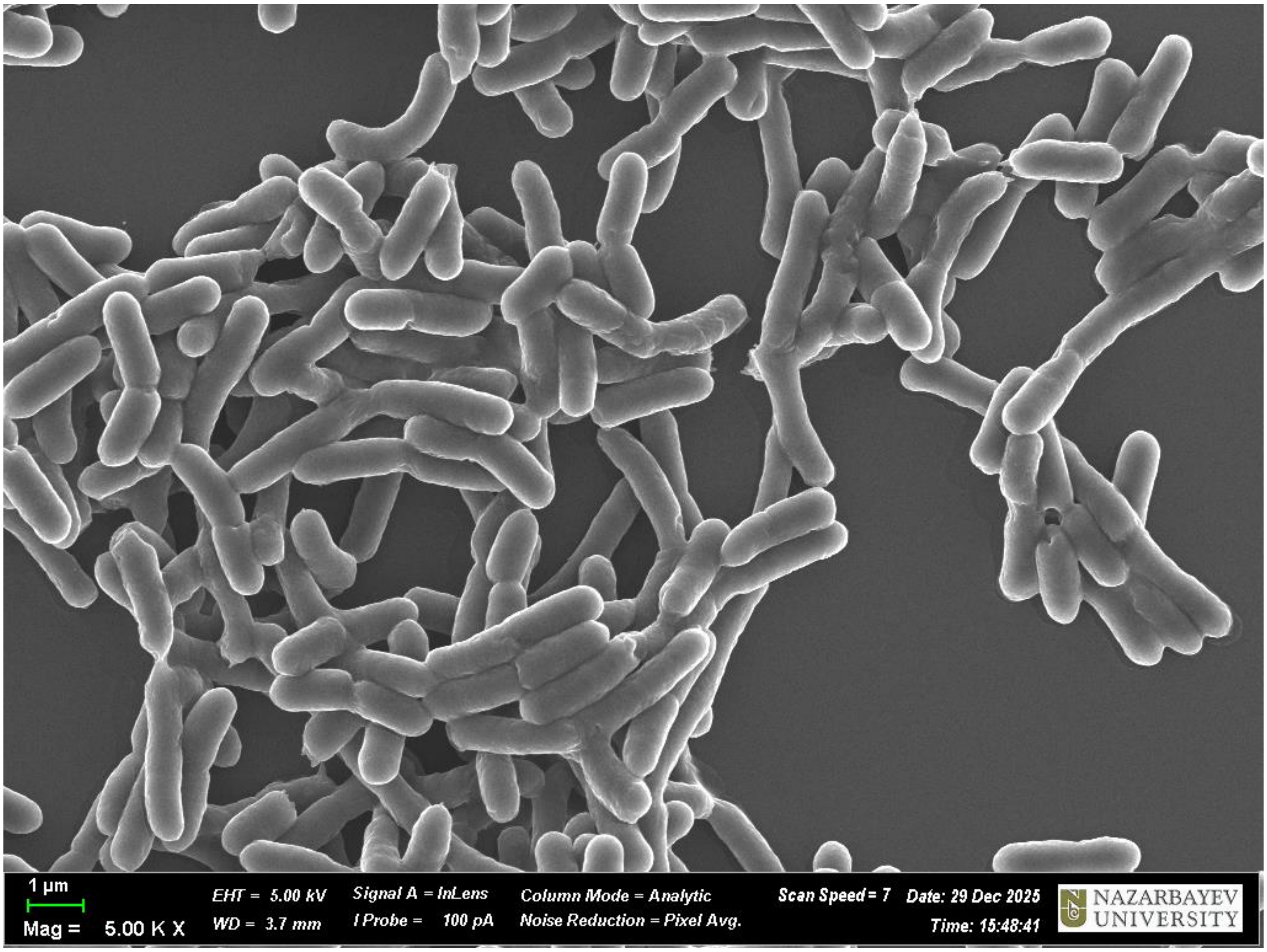

Figure S6. SEM of strain JSM-10, dense bacterial aggregation.

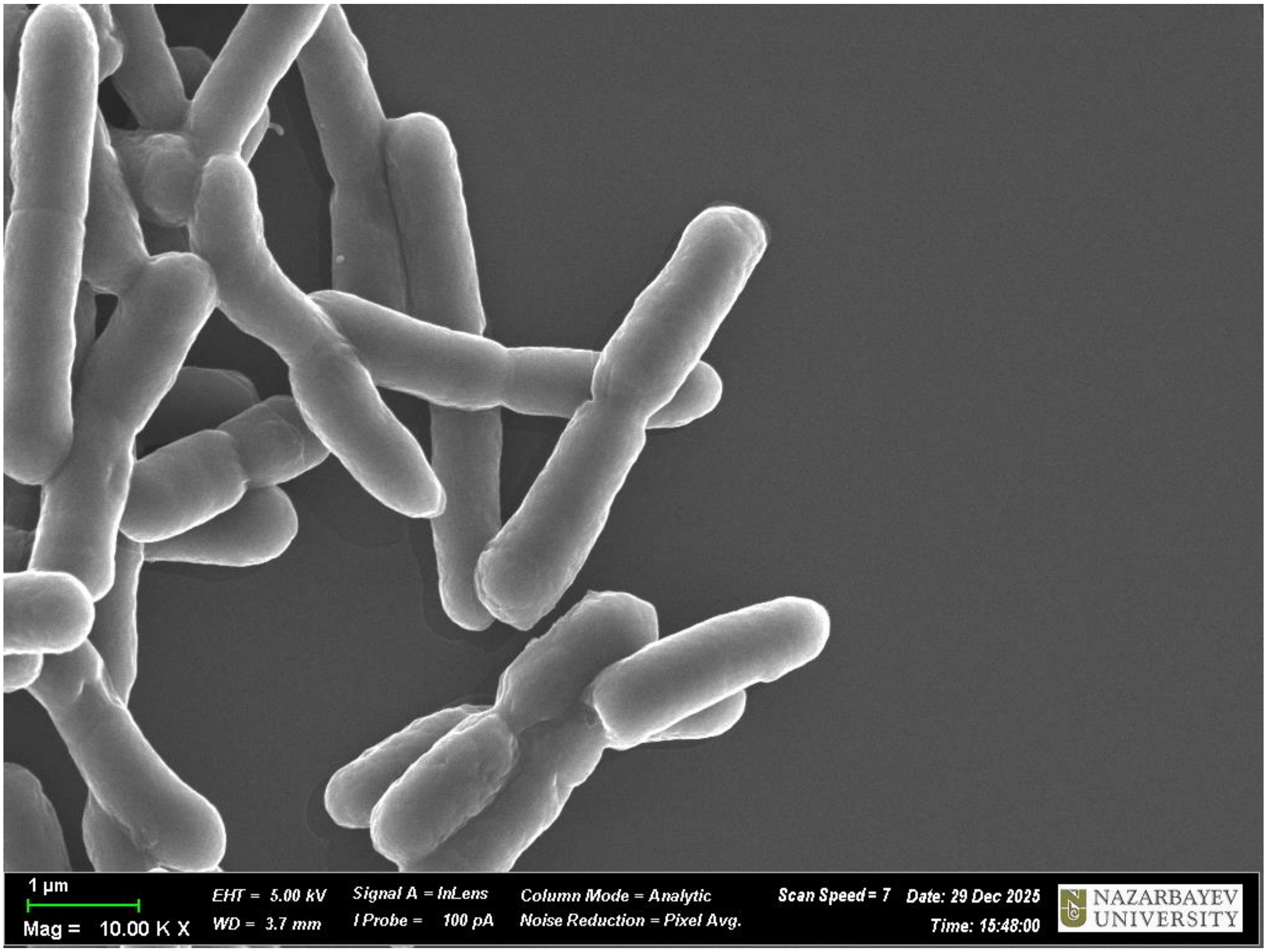

Figure S7. SEM of strain JSM-10, high-magnification image of a dividing cell.

TEM:

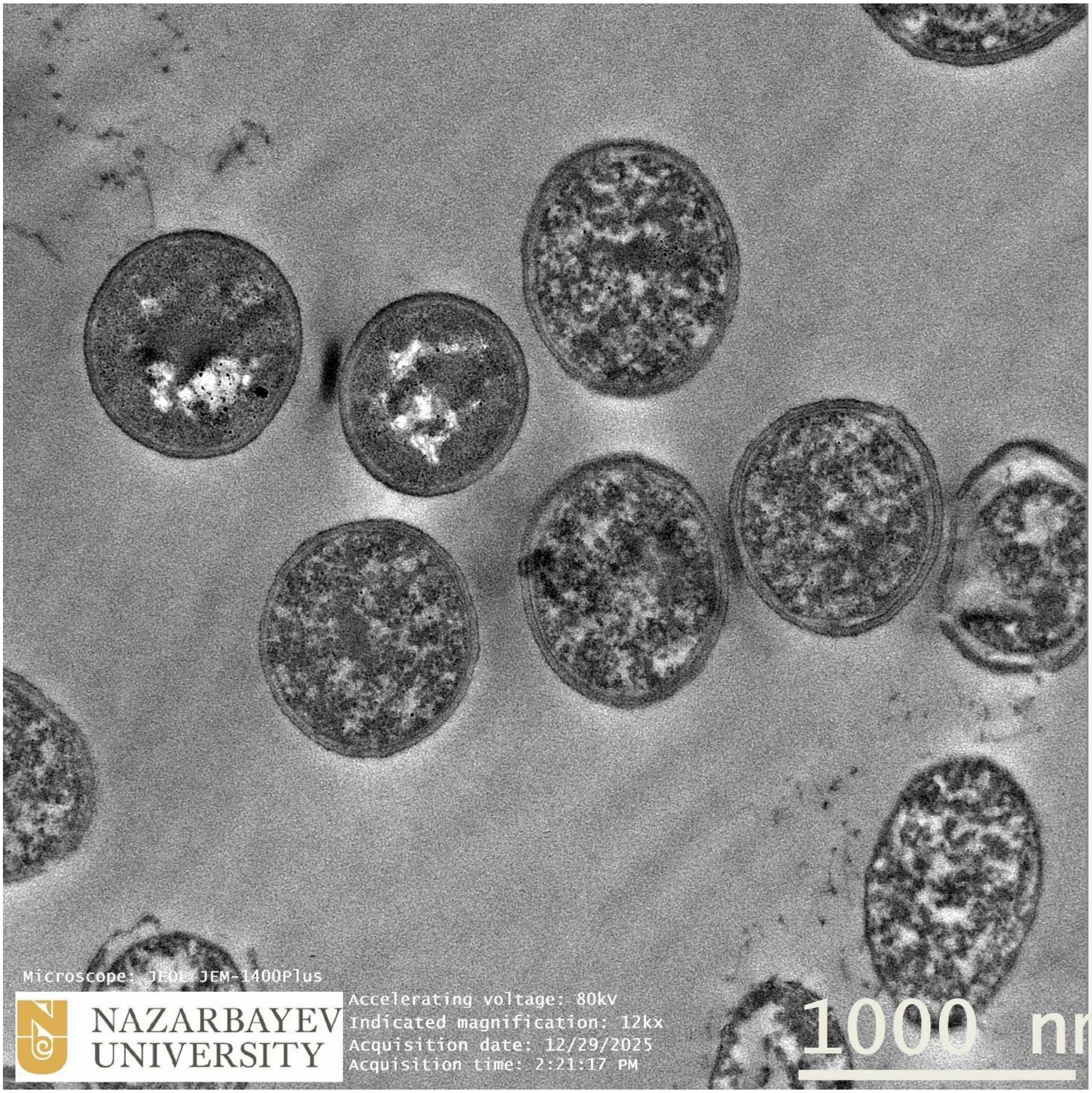

Figure S8. TEM of strain JSM-10 as shown in **Figure 2f (uncropped original image).**

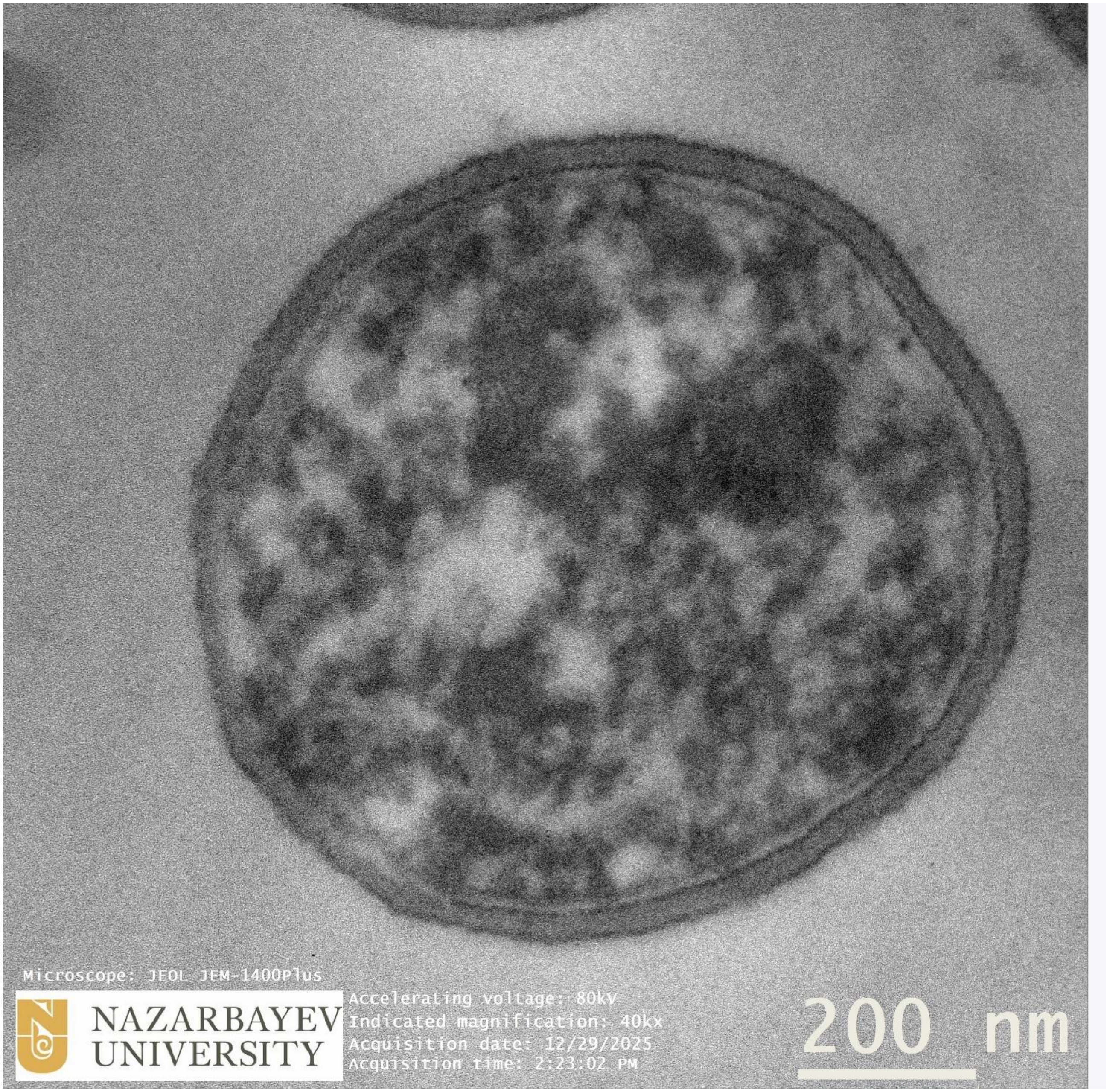

Figure S9. TEM of strain JSM-10 as shown in **Figure 2g (uncropped original image).**

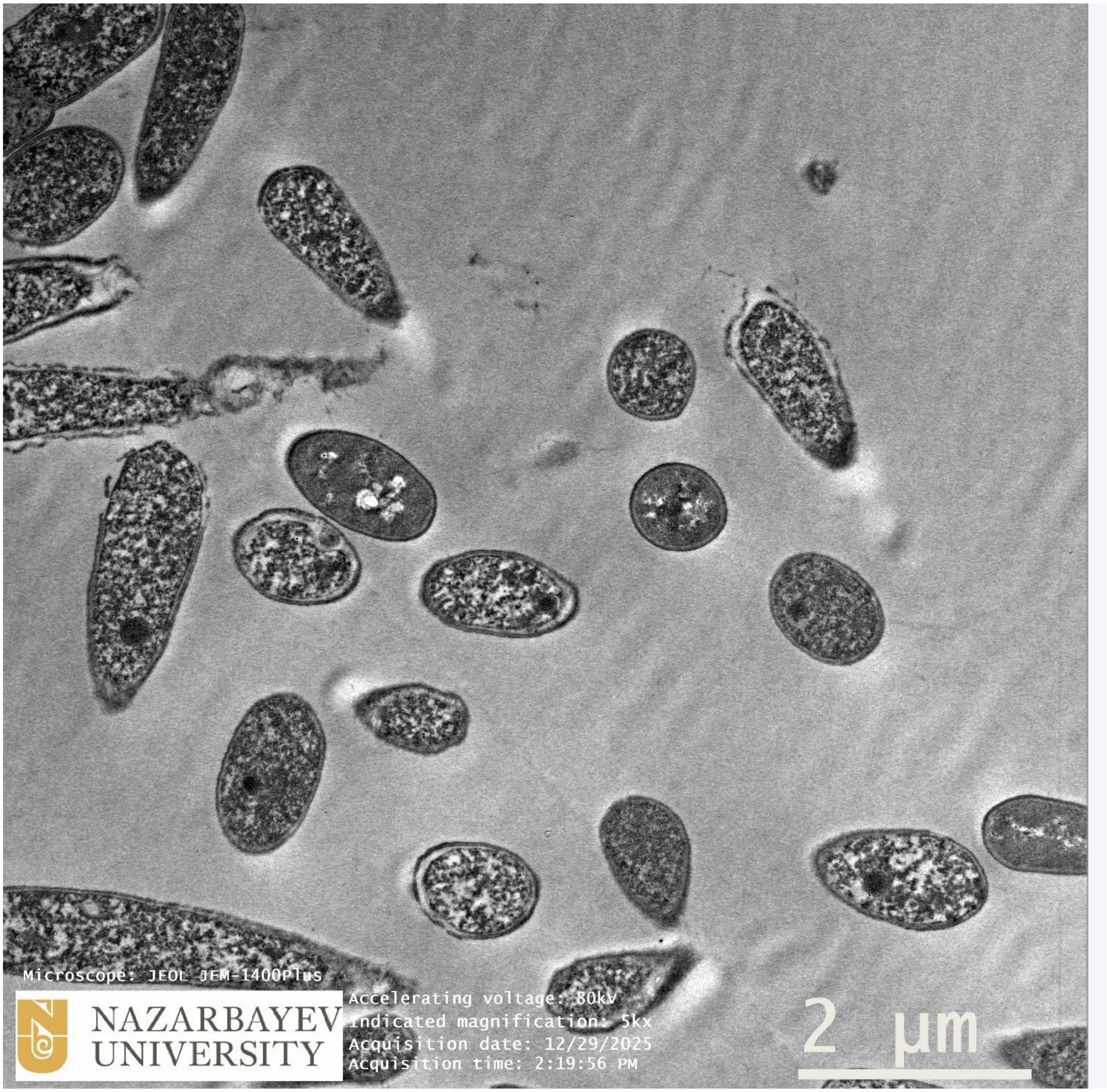

Figure S10. TEM of strain JSM-10, elongated cells displaying structural heterogeneity.

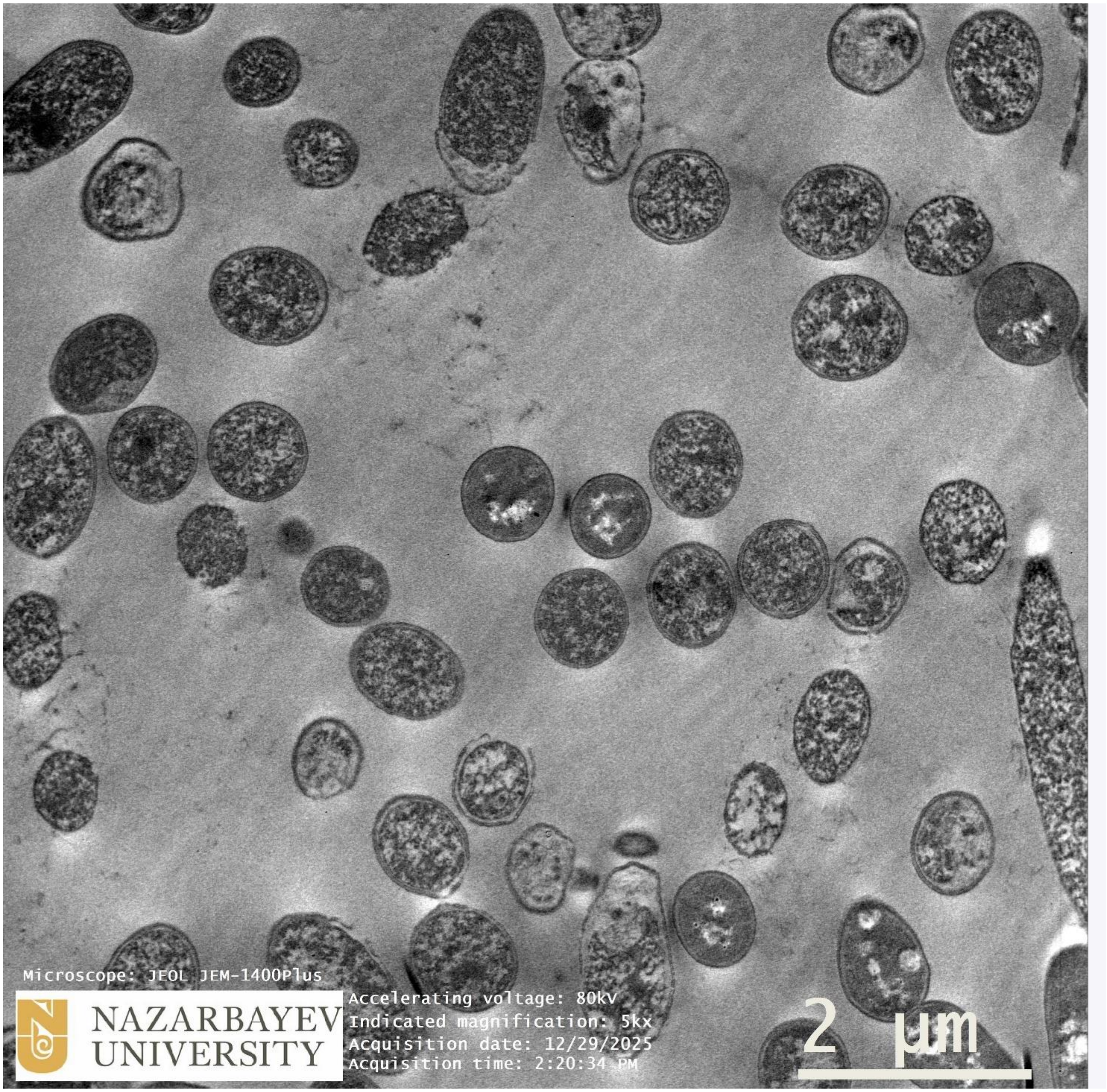

Figure S11. TEM of strain JSM-10, detailed view of intracellular components and cellular morphology.

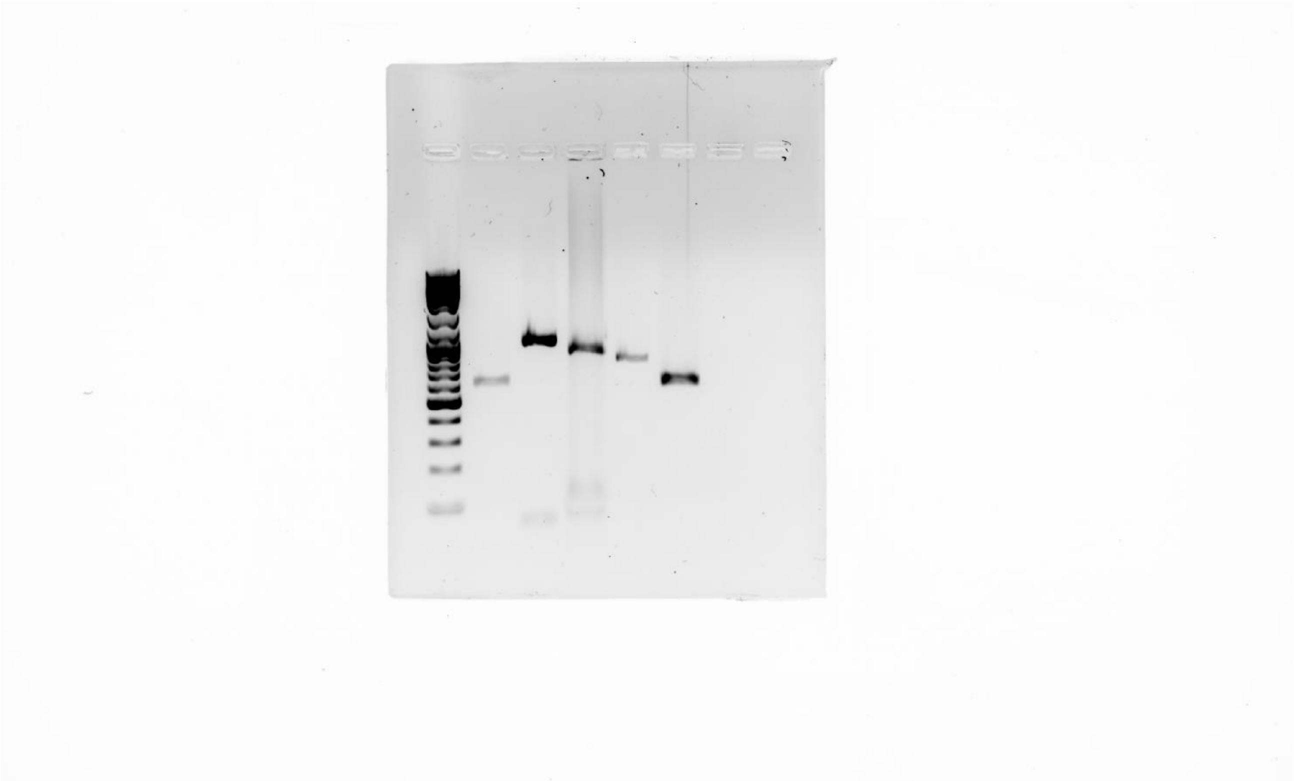

Figure S12. Molecular detection of genes associated with plant growth promotion and stress tolerance as shown in Figure 8 **(uncropped original image).**
